# Supplementary material for: Impact of COVID-19 on outpatient appointments in children and young people in England: an observational study
Source: BMJ Open. 2022 Aug 8;12(8):e060961. doi: 10.1136/bmjopen-2022-060961 (PMC9364042; doi:10.1136/bmjopen-2022-060961)

BMJ Publishing Group Limited (BMJ) disclaims all liability and responsibility arising from any reliance placed on this supplemental material which has been supplied by the author(s)

Supplementary material

Figure S1. All scheduled appointments since Jan 2018 by sex for three age groups. Poisson regression demonstrated a negligible monthly linear trend 2017-2019.

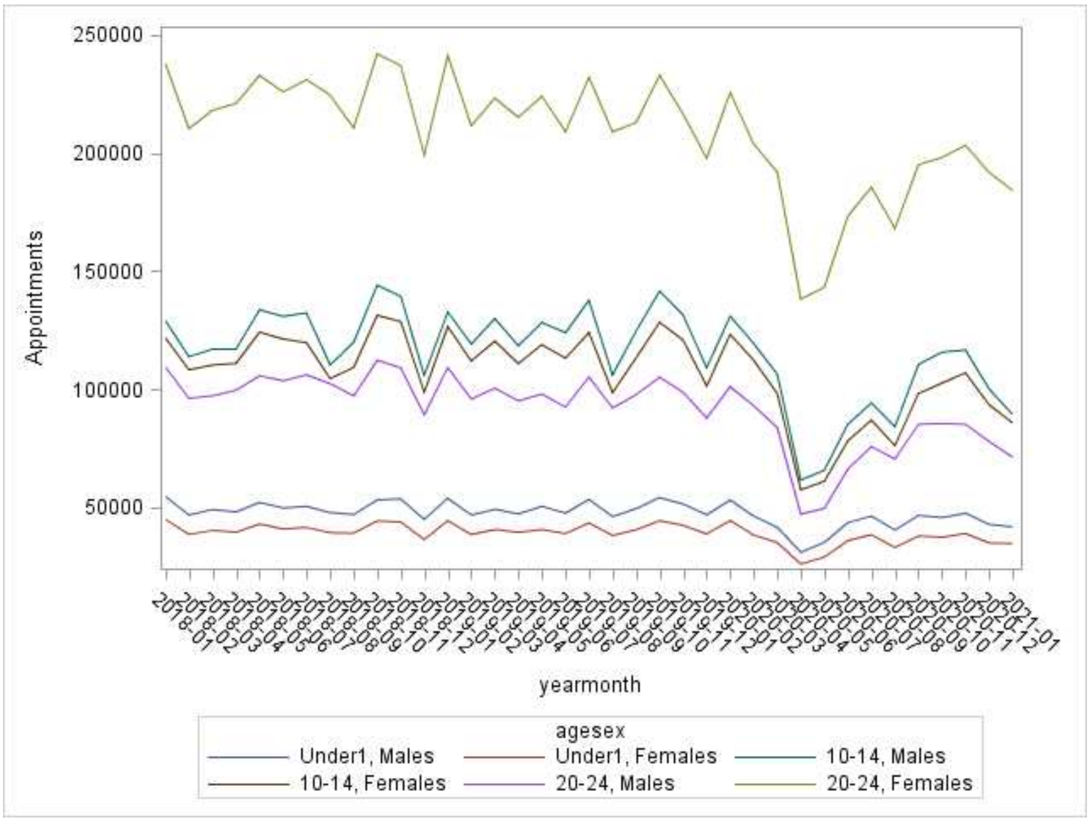

Figure S2. All appointments, all ages, split by whether the specialty was surgical

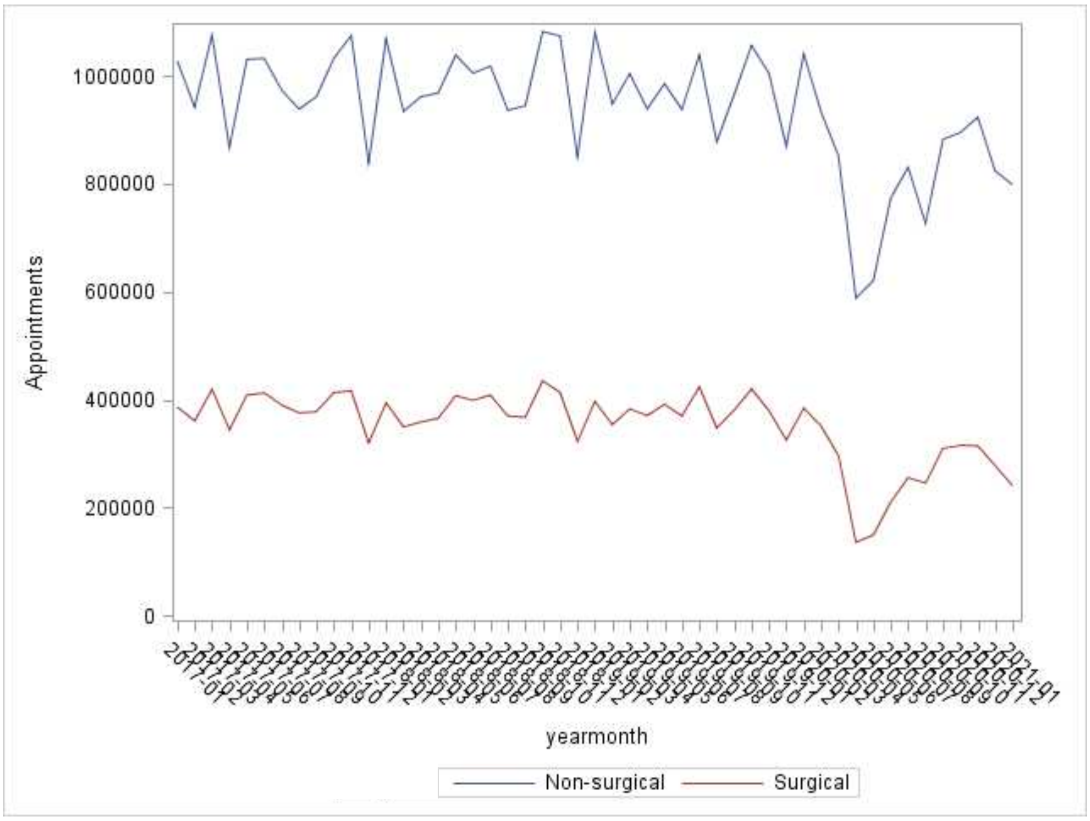

Figure S3. Trends in first appointments (both face-to-face and telephone) by IMD fifth; IMD 1 is least deprived, IMD 5 most deprived

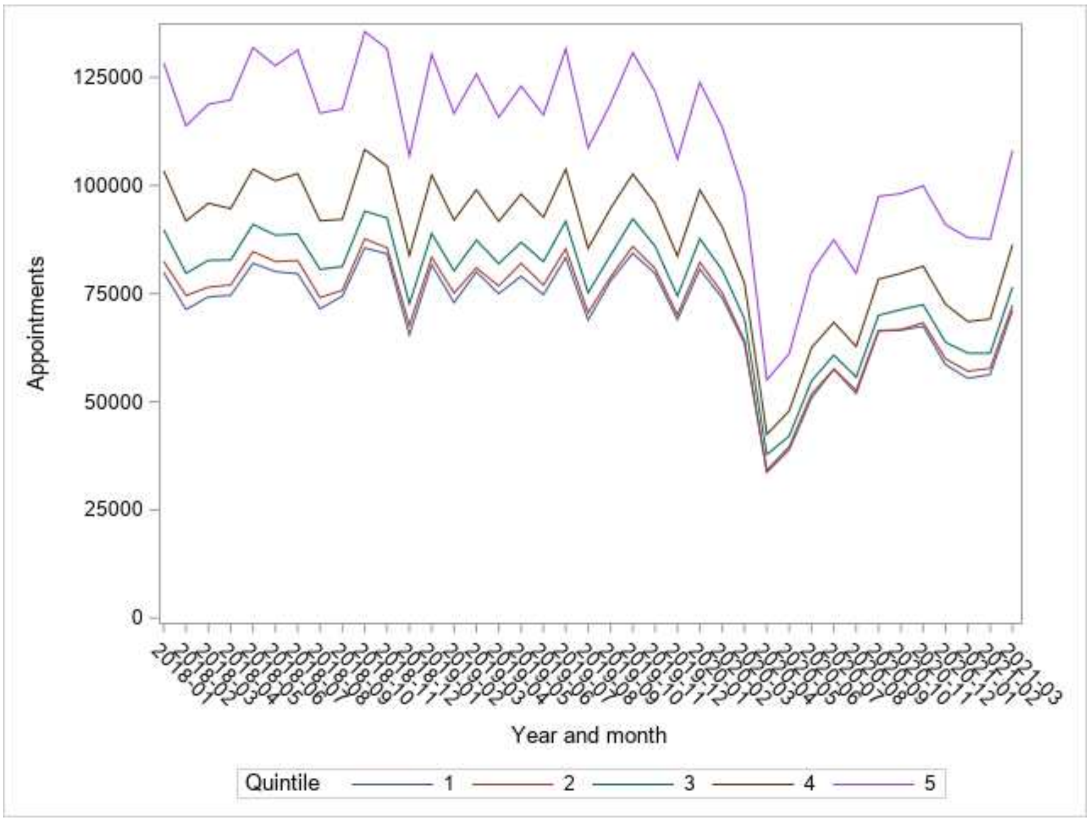

Figure S4. Trends in follow up appointments (both face-to-face and telephone) by IMD fifth; IMD 1 is least deprived, IMD 5 most deprived

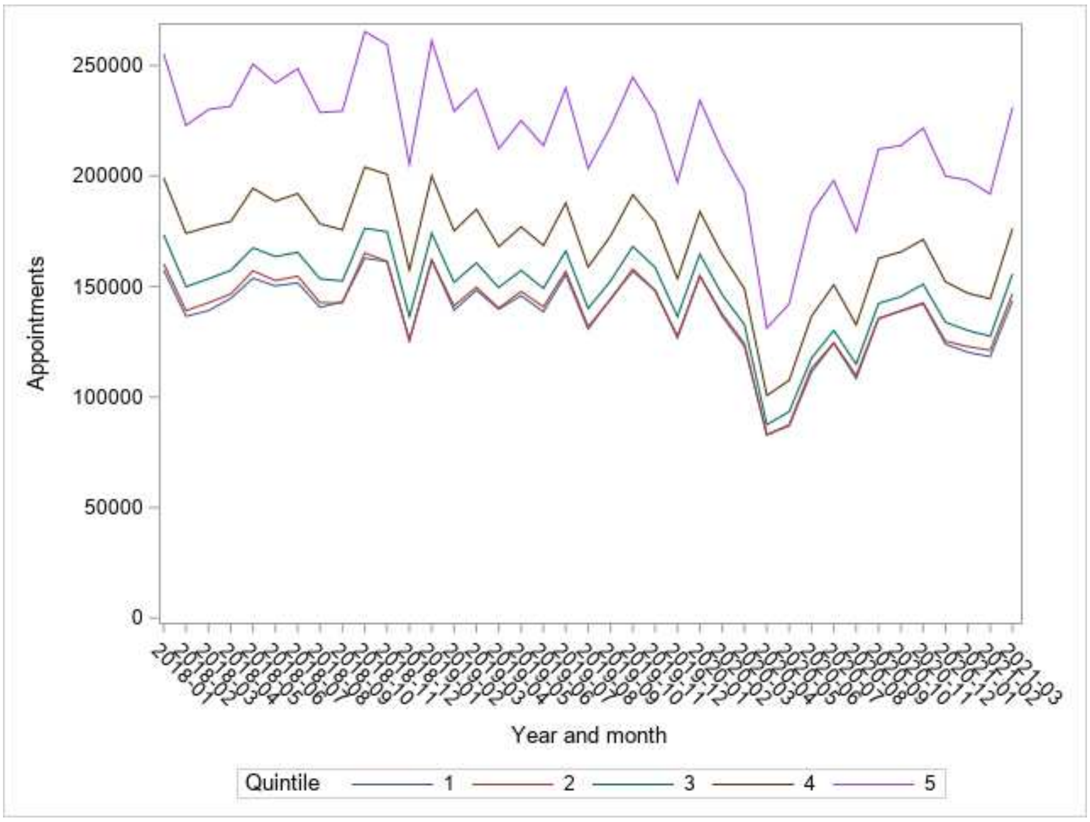

Table S1. Total number of first and follow up appointments face-to-face (F2F) or by telephone, stratified by age and period.

	Jan 2017 to Dec 2019 average				Oct 2020 to Feb 2021 average*			
Age (years)	First appt F2F	First appt by phone	Follow up appt F2F	Follow up appt by phone	First appt F2F	First appt by phone	Follow up appt F2F	Follow up appt by phone
Under-1	595004	9254	459800	22274	427898	59443	335055	91541
1-4	875210	11806	1490240	47186	467160	115895	854808	365760
5-9	893452	20157	1904357	61848	469174	123935	1084465	457184
10-14	862405	30844	1879837	65696	458642	128777	1093129	450506
15-19	916698	28264	2115660	68580	528163	144721	1279882	452514
20-14	1328937	30532	2428495	69945	831865	190481	1548571	447965
Total	5471706	130857	10278389	335529	3182902	763252	6195910	2265470

* Oct 2020 to Feb 2021 is given because the first wave of covid-19 had ended, and the system had stabilised

Figure S5. Increase compared with three-year average for 1st phone appointments

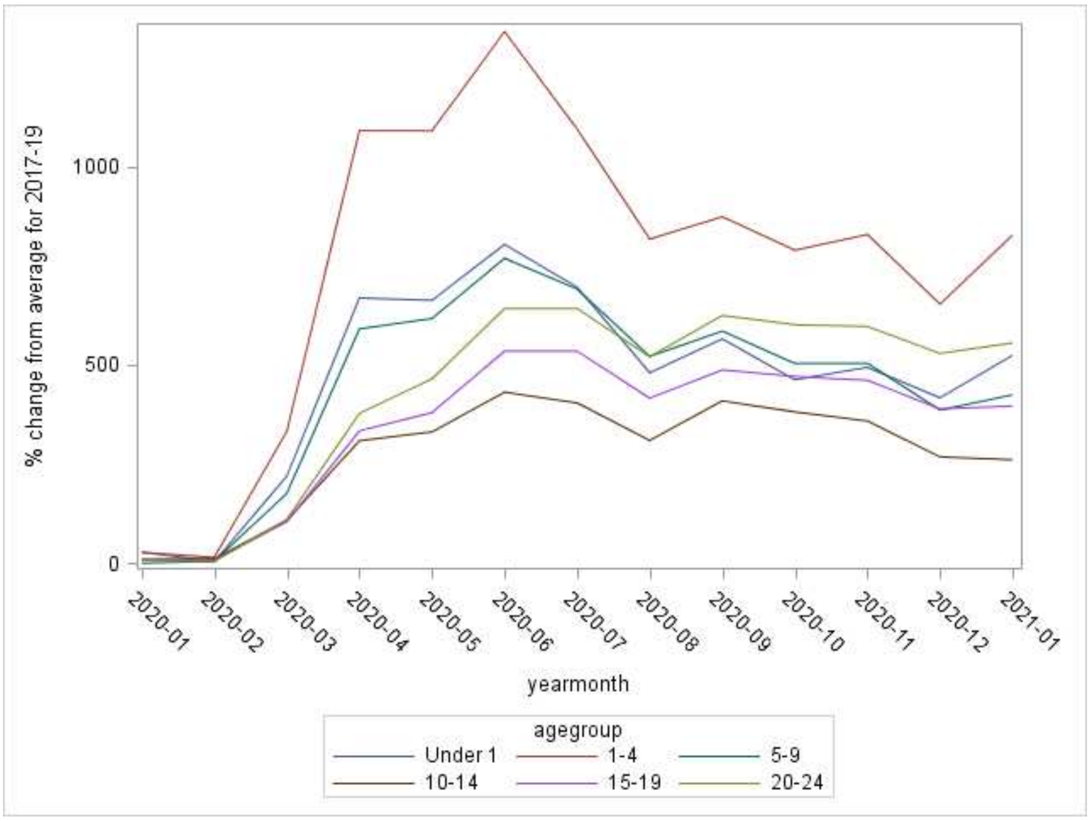

Figure S6. Attendance rates for the under-1s, comparing F2F with phone appts

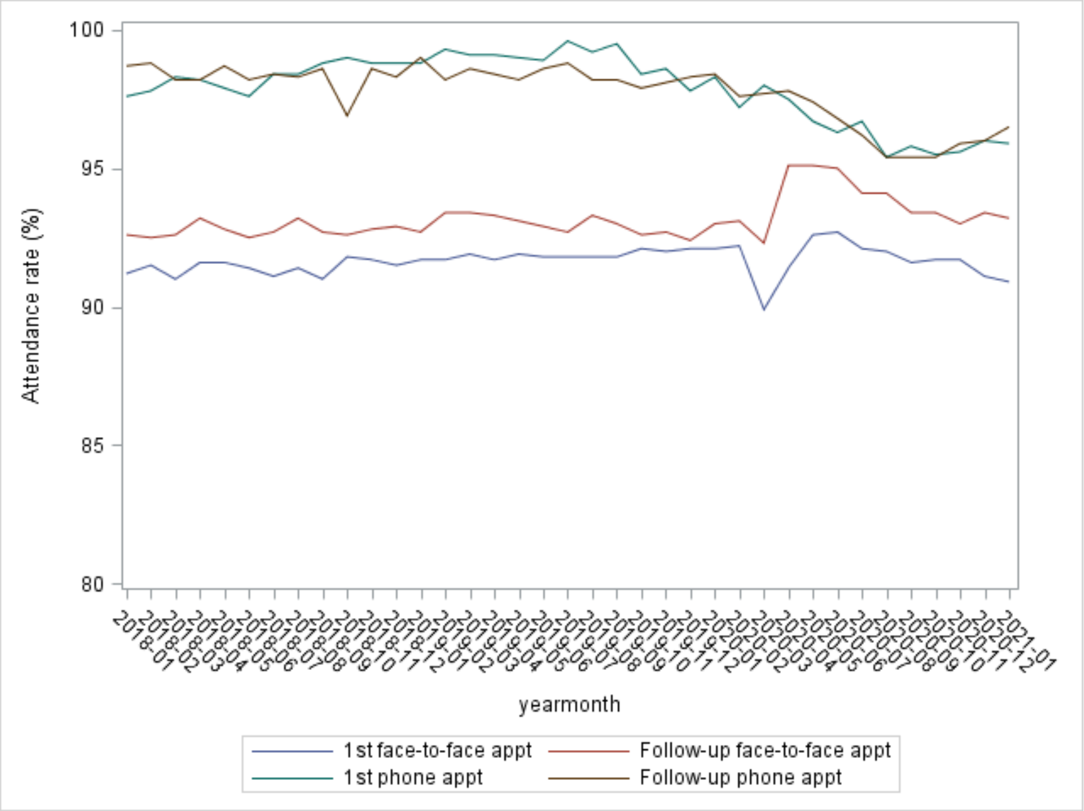

The pattern is repeated for the other age groups. For example, here are the attendance rates for those aged 20-24:

Figure S7. Attendance rates for the 20-24s, comparing F2F with phone appts

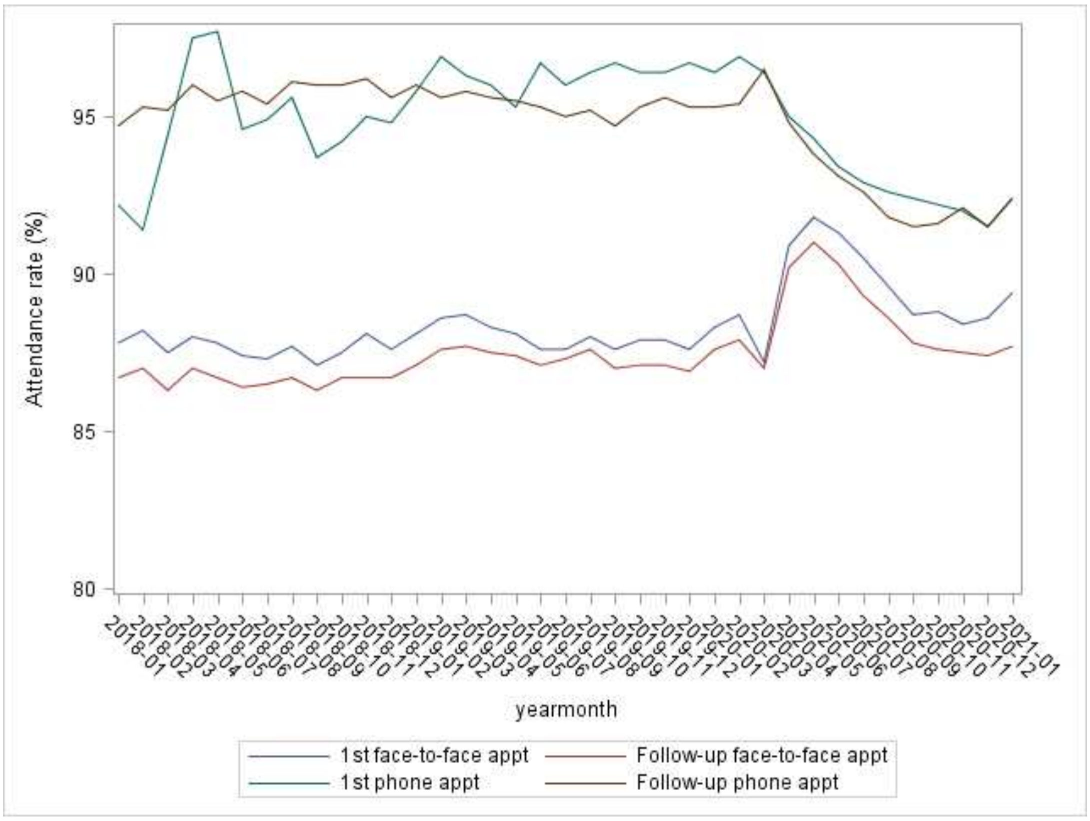

Supplement: Supplementary data [file bmjopen-2022-060961supp001.pdf]
