# Supplementary material for: Impact of COVID-19 on outpatient appointments in children and young people in England: an observational study
Source: BMJ Open. 2022 Aug 8;12(8):e060961. doi: 10.1136/bmjopen-2022-060961 (PMC9364042; doi:10.1136/bmjopen-2022-060961)
Supplement: Author's manuscript [file bmjopen-2022-060961.draft_revisions.pdf]

BMJ Open is committed to open peer review. As part of this commitment we make the peer review history of every article we publish publicly available.

When an article is published we post the peer reviewers' comments and the authors' responses online. We also post the versions of the paper that were used during peer review. These are the versions that the peer review comments apply to.

The versions of the paper that follow are the versions that were submitted during the peer review process. They are not the versions of record or the final published versions. They should not be cited or distributed as the published version of this manuscript.

BMJ Open is an open access journal and the full, final, typeset and author-corrected version of record of the manuscript is available on our site with no access controls, subscription charges or pay-per-view fees (http://bmjopen.bmj.com).

If you have any questions on BMJ Open's open peer review process please email info.bmjopen@bmj.com

BMJ Open

Impact of Covid-19 on outpatient appointments in children and young people in England: an observational study

Journal:	*BMJ Open*
Manuscript ID	bmjopen-2022-060961
Article Type:	Original research
Date Submitted by the Author:	12-Jan-2022

Complete List of Authors:	Bottle, Alex; Imperial College London, Department of Primary Care and Public Health<br>Neale, Francesca; Imperial College London, Department of Primary Care and Public Health<br>Foley, Kimberley; Imperial College London, Department of Primary Care and Public Health<br>Viner, Russell; UCL Great Ormond Street Institute of Child Health Population Policy and Practice<br>Kenny, Simon; NHS England and NHS Improvement London; University of Liverpool, Institute of Systems, Molecular and Integrative Biology<br>Aylin, Paul; Imperial College London, Department of Primary Care and Public Health<br>Saxena, Sonia; Imperial College London, Department of Primary Care and Public Health<br>Hargreaves, Dougal; Imperial College London, Department of Primary Care and Public Health

Keywords:	PAEDIATRICS, COVID-19, PUBLIC HEALTH

SCHOLARONE™
Manuscripts

I, the Submitting Author has the right to grant and does grant on behalf of all authors of the Work (as defined in the below author licence), an exclusive licence and/or a non-exclusive licence for contributions from authors who are: i) UK Crown employees; ii) where BMJ has agreed a CC-BY licence shall apply, and/or iii) in accordance with the terms applicable for US Federal Government officers or employees acting as part of their official duties; on a worldwide, perpetual, irrevocable, royalty-free basis to BMJ Publishing Group Ltd ("BMJ") its licensees and where the relevant Journal is co-owned by BMJ to the co-owners of the Journal, to publish the Work in this journal and any other BMJ products and to exploit all rights, as set out in our licence.

The Submitting Author accepts and understands that any supply made under these terms is made by BMJ to the Submitting Author unless you are acting as an employee on behalf of your employer or a postgraduate student of an affiliated institution which is paying any applicable article publishing charge ("APC") for Open Access articles. Where the Submitting Author wishes to make the Work available on an Open Access basis (and intends to pay the relevant APC), the terms of reuse of such Open Access shall be governed by a Creative Commons licence – details of these licences and which Creative Commons licence will apply to this Work are set out in our licence referred to above.

Other than as permitted in any relevant BMJ Author's Self Archiving Policies, I confirm this Work has not been accepted for publication elsewhere, is not being considered for publication elsewhere and does not duplicate material already published. I confirm all authors consent to publication of this Work and authorise the granting of this licence.

For peer review only

Impact of COVID-19 on outpatient appointments in children and young people in England: an observational study

Alex Bottle[1], Francesca K Neale[1], Kimberley A Foley[1], Russel Viner[2], Simon E Kenny[3,4,5], Paul Aylin[1], Sonia Saxena[1], Dougal S Hargreaves[1]

1. Department of Primary Care and Public Health, Imperial College London School of Public Health, London, UK
2. Population, Policy and Practice Research Programme, UCL Institute of Child Health, London, UK
3. NHS England and NHS Improvement, London, UK
4. Institute of Systems, Molecular and Integrative Biology, University of Liverpool, Liverpool, UK
5. Department of Paediatric Surgery, Alder Hey in the Park, Liverpool, UK

Correspondence to:
Dr Francesca Neale, Department of Primary Care and Public Health, Imperial College London School of Public Health, London, W6 8RP, UK; 020 7594 3368; francesca.neale1@nhs.net

Keywords:
Outpatient appointments
Children
Young people
Health service use
Covid-19

Word count: 2901 (excluding title page, references, figures and tables)

For peer review only - http://bmjopen.bmj.com/site/about/guidelines.xhtml

Impact of COVID-19 on outpatient appointments in children and young people in England: an observational study

Abstract

Objectives: To describe the impact of the Covid-19 pandemic on outpatient appointments for children and young people.

Setting: All National Health Service (public) hospitals in England.

Participants: All people in England aged <25 years.

Outcome measures: Outpatient department attendance numbers, rates and modes (face-to-face vs telephone) by age group, sex, and socio-economic deprivation.

Results: Compared with the average for January 2017 to December 2019, there was a 3.8 million appointment shortfall (23.5%) for the under-25 population in England between March 2020 and February 2021, despite a total rise in phone appointments of 2.6 million during that time. This was true for each age group, sex and deprivation fifth, but there were smaller decreases in face-to-face and total appointments for babies under 1 year. For all ages combined, around one in six first and one in four follow-up appointments were by phone in the most recent period. The proportion of appointments attended was high, at over 95% for telephone and over 90% for face-to-face appointments for all ages.

Conclusions: Covid-19 led to a dramatic fall in total outpatient appointments and a large rise in the proportion of those appointments conducted by telephone. The impact that this has had on patient outcomes is still unknown. The differential impact of Covid-19 on outpatient

For peer review only - http://bmjopen.bmj.com/site/about/guidelines.xhtml

activity in different socio-demographic groups may also inform design of paediatric outpatient services in the post-Covid period.

Strengths and Limitations

- This is the largest study of outpatient appointment patterns in children and young people before and after March 2020 in England.
- It benefits from national data collected in a standardised way. Data quality in such databases for administrative items like dates, age and sex is very high.
- We were unable to analyse patterns by diagnosis due to very low levels of recording.
- We examined several patient subgroups, but future work could look at patterns by hospital trust and specialty.

Introduction

The Covid-19 pandemic has led to profound disruption to health services globally as treatment of Covid-19 infections was prioritised over some groups felt to be at lower risk, including children and young people. In the United Kingdom (UK), strict lockdown measures were introduced in March 2020, November 2020 and January 2021. The disruptions to daily life and health services, including the temporary closure of some paediatric wards and emergency departments, led to changes to health seeking behaviour and health service use. Reductions in emergency department attendance and emergency admissions have been documented in both children (1–8) and adults (9). A national retrospective cohort study in Scotland described reductions of almost two-thirds in paediatric unscheduled primary care presentations and half of emergency secondary care presentations, without any change in Paediatric Intensive Care Unit admissions or mortality (6). Falls in urgent care contacts compared with previous years led to speculation that children and young people's (CYP) health might deteriorate if serious conditions were missed or if they experienced delays in diagnosis of chronic disease. However, studies have found no significant impact on route to diagnosis and severity of presentation for type 1 diabetes and new diagnosed cancers, as well as no impact on overall outcomes for asthma, pyloric stenosis and appendicitis (10–14).

There have also been concerns that some CYP were unable to access specialist services in hospitals. For example, a study of radiology outpatient activity at a tertiary children's hospital from March 2020-March 2021 showed a reduction of 27.3% (15). The British Medical Association estimated that across all ages in England between April and June 2020 there were between 2.47 million and 2.6 million fewer first outpatient attendances than expected (16). Similarly, The Health Foundation reported a fall of 4.4 million outpatient appointments in England in May 2020 compared with May 2019 and 4 million fewer GP

For peer review only - http://bmjopen.bmj.com/site/about/guidelines.xhtml

referrals to outpatient appointments between January and October 2020 compared with the same period in 2019, although referrals for suspected cancer had recovered to pre-pandemic levels by October 2020 (17). However, no detailed analysis has been published of paediatric outpatient activity, where growing concerns had already been raised about the equity of access to specialist care pre-Covid (18).

Telephone and video consultations have been used prior to the Covid-19 pandemic in many specialties, including paediatrics, with advantages including less time wasted between appointments, no associated costs, fewer late arrivals and, since the Covid-19 pandemic, patients are understandably anxious about the risk of catching Covid-19 in hospital. Disadvantages of remote consultations include missing non-verbal cues, the exclusion of those without access to technology, the inability to examine the patient, and that many doctors have not been trained to conduct telephone consultations (19–21). However, it is unknown to what extent remote paediatric consultations were used during the Covid-19 pandemic.

We make use of England's national hospital administrative database, Hospital Episodes Statistics (HES), to describe trends in outpatient appointment numbers, rates and modes (face-to-face vs telephone) in CYP by age group, sex, and socio-economic deprivation between January 2017 and January 2021 in England.

Methods

Data

We extracted HES data for outpatient appointments between 1st Jan 2017 and 28th Feb 2021 and for patients aged under 25. Records were excluded if either the patient or the hospital cancelled the appointment or in the rare cases when the age or sex was invalid. Area-level Index of Multiple Deprivation (IMD) was attached to HES for each patient and turned into population-weighted fifths so that nationally, for all ages combined, each fifth contained equal population. The ATENTYPE field distinguishes between first and follow-up appointments, with separate values for face-to-face, telephone and not specified; we excluded records for 'not specified' when comparing face-to-face and telephone. It also states whether the patient attended. The term telephone consultations refers to both telephone and video consultations as no separate value for video consultations is available. The pre-pandemic period was defined as 1st Jan 2017 to 29th Feb 2020; the pandemic period was defined as 1st March 2020 onwards.

Statistical analysis

Appointment counts and attendance rates (i.e., proportion of appointments that the patient attended) were plotted by month and patient subgroup, including by age (under-1, 1-4, 5-9, 10-14, 15-19 and 20-24). For ease of comparison, changes in appointment numbers were calculated for the 12-month period from 1st Mar 2020 to 28th Feb 2021 rather than to the end of Mar 2021. This was done by comparing total counts with age-matched yearly averages across the three-year period 1st Jan 2017 to 31st Dec 2019 rather than use time-series predictions, for example, as no clear monthly trends were noted during that time.

For peer review only - http://bmjopen.bmj.com/site/about/guidelines.xhtml

Changes were expressed in absolute terms and as percentages. Due to the descriptive rather than hypothesis-driven nature of our study and the very large numbers involved, no statistical tests were performed.

Ethics

We had approval from the Secretary of State and the Health Research Authority under Regulation 5 of the Health Service (Control of Patient Information) Regulations 2002 to hold confidential data and analyse them for research purposes (CAG ref 15/CAG/0005). We have approval to use them for research and measuring quality of delivery of healthcare, from the London - South East Ethics Committee (REC ref 20/LO/0611).

Patient and Public Involvement

There was no patient or public involvement in this research.

Results

For CYP aged under 25 years, the overall fall in outpatient appointments for the 12-month period Mar 2020 to Feb 2021 compared with the means for the previous three years was over 3.8 million, a 23.5% fall, with the biggest percentage fall in the 5-9s and the smallest percentage change in under 1s (Table 1). This was despite a total rise in phone appointments of 2.6 million.

Table 1. Changes in numbers of appointments by age group for Mar 2020 to Feb 2021 compared with yearly average across the period Jan 2017 to Dec 2019

Age (years)	First F2F appt	Follow-up F2F appt	First phone appt	Follow-up phone appt	Total (% change)
Under 1	-167,106 (-28.1)	-124,745 (-27.1)	+50,189 (+542.4)	+69,267 (+311.0)	-172,395 (-15.9%)
1-4	-408,050 (-46.6)	-635,432 (-42.6)	+104,189 (+881.6)	+318,574 (+675.2)	-620,819 (-25.6%)
5-9	-424,378 (-47.5)	-819,892 (-43.1)	+103,778 (+514.9)	+395,336 (+639.2)	-745,056 (-25.9%)
10-14	-403,763 (-46.8)	-786,708 (-41.8)	+97,933 (+317.5)	+384,810 (+585.7)	-707,728 (-24.9%)
15-19	-388,535 (-42.4)	-835,778 (-39.5)	+116,457 (+412.0)	+383,934 (+559.8)	-723,922 (-23.1%)
20-24	-497,072 (-37.4)	-879,924 (-36.2)	+159,949 (+523.9)	+378,020 (+540.5)	-839,027 (-21.7%)
Total	-2,288,804 (-41.8)	-4,082,479 (-39.7)	+632,395 (+483.3)	+1,929,941 (+575.2)	-3,808,947 (-23.5%)

F2F = Face-to-face; "first" and "follow-up" are defined by HES

Total outpatient appointment numbers increased from May 2020 but had not reached pre-pandemic levels by Mar 2021 (Figure 1). This was true for each age group and sex (Appendix Figure S1) and for surgical and non-surgical specialties (Appendix Figure S2). The same

For peer review only - http://bmjopen.bmj.com/site/about/guidelines.xhtml

pattern was observed for each IMD fifth (Appendix Figure S3, S4), which was unchanged since Mar 2020. To illustrate, Figure 2 shows this for the under-1s and those aged 20-24: for all age groups, the less-affluent areas had the most appointments.

Phone appointments increased by 15% from Mar 2020 to Feb 2021 for both first and follow-up visits. Table 2 compares pre-pandemic (Jan 2017 to Dec 2019) averages with Oct 2020 to Feb 2021 averages. The largest relative changes were seen among under 1s with tripling of the proportion of first appointments performed by phone (1.5% to 4.6%) and almost doubling of the proportion of follow-up appointments performed by phone (10.5% to 20.0%). The biggest absolute percentage rises were for the 10-14s (Table 2).

For peer review only

Table 2. Percentage of first and follow-up appointments that are by telephone, stratified by age and period

	Jan 2017 to Dec 2019 average		Oct 2020 to Feb 2021 average*	
Age (years)	First appt % by phone	Follow-up appt % by phone	First appt % by phone	Follow-up appt % by phone
Under-1	1.5	10.5	4.6	20.0
1-4	1.3	16.5	3.1	26.3
5-9	2.2	17.2	3.1	25.8
10-14	3.5	18.8	3.4	26.0
15-19	3.0	19.8	3.1	23.8
20-24	2.2	18.5	2.8	22.6
Total	2.3	17.3	3.2	24.5

* Oct 2020 to Feb 2021 is given because the first wave of covid-19 had ended and the system had stabilised

For all ages combined, around one in six first and one in four follow-up appointments were by phone in the most recent period. Appendix Figure S5 shows the rise in phone appointments by age relative to the pre-pandemic period.

Proportions of appointments attended

Attendance was high, at over 95% for telephone and over 90% for face-to-face appointments for all ages. There were slight falls in the former and slight rises in the latter from March 2021 (Appendix Figure S6, S7). Attendance and patterns did not differ by sex,

For peer review only - http://bmjopen.bmj.com/site/about/guidelines.xhtml

with the exception that in the 20-24s, attendance was higher for women than for men, a difference driven by face-to-face appointments.

Discussion

Summary of results

In England's NHS, CYP outpatient appointments fell sharply in March 2020 and had still not recovered to pre-pandemic levels in March 2021. In total, the 12 months from March 2020 saw 3.8 million appointments fewer (23.5%) than expected from the average for 2017-2019. The fall affected all ages, sexes, specialties (categorised as surgical or non-surgical), and deprivation quintiles. The huge rise in telephone use did not compensate for the bigger fall in face-to-face visits. Around one in five appointments were by phone for this population at the end of the study period.

Attendance rates were high and were higher for phone appointments than for face-to-face ones; the only sex difference was that women aged 20-24 had higher rates than men; for first phone appointments, this sex difference disappeared and was much reduced for follow-ups. In contrast to research showing persistent and systemic child health inequalities in the UK (22), there was little variation in appointment numbers by Index of Multiple Deprivation quintile.

Findings compared with previous studies

Our findings fit with other national research that demonstrated large reductions in secondary care health service use since March 2020 (6,9), including reduced outpatient activity for all age groups (17) Whereas total suspected cancer referrals returned to pre-pandemic levels by Oct 2020, we found that CYP outpatient activity had not returned to pre-pandemic levels at any point by Mar 2021.

Since the Covid-19 pandemic, there has been a significant rise in virtual consultations in CYP outpatient departments globally. A large global survey of healthcare professionals caring for CYP with diabetes found that 50% had switched to virtual consultations (23), 36 of 38 surveyed North American Paediatric surgical units had switched to virtual outpatient appointments (24), a global survey of paediatric neurologists reported a large increase in outpatient virtual consultations (25) and a multicentre longitudinal observational study of Paediatric orthopaedic trauma in London also described greater use of outpatient virtual consultations (26).

Previous studies have also found that in CYP outpatient clinics, telephone appointment attendance is higher than face-to-face appointment attendance (27,28). Studies have shown high levels of patient satisfaction with telephone appointments during the Covid-19 pandemic (29–31) and high levels of satisfaction amongst HCPs providing a remote CYP neurology outpatient service (32). However, concerns have been raised about the quality of care provided in remote consultations, especially for first appointments (33,34). Research is emerging on how remote CYP outpatient appointments should best be routinely integrated into post-Covid services (35–37).

For peer review only - http://bmjopen.bmj.com/site/about/guidelines.xhtml

The extent to which the fall in CYP outpatient activity can be attributed to a decrease in health seeking behaviour is unknown. A retrospective national cohort study into the decrease of Paediatric secondary care emergency presentations hypothesised that, as there were no increases in clinical severity scores, Paediatric Intensive Care Unit (PICU) admissions or mortality, caregivers were often appropriately able to use a higher threshold for seeking medical attention (6). Another study on this topic also found that 93.5% of CYP presenting to secondary care during a two-week period in April-May 2020 were not felt to have had a delay in their presentation (4). Further research is required to establish the extent to which the fall in CYP outpatient activity since March 2020 is a result of changes in health seeking behaviour, although it is unlikely to have played as large a role as in emergency presentations as we observed a dramatic fall in both first and follow up appointments.

Strengths and limitations

This is the largest study of outpatient appointment patterns in CYP before and after March 2020 in England. It benefits from national data collected in a standardised way. Data quality in such databases for administrative items like dates, age and sex is very high, but we were unable to analyse patterns by diagnosis due to very low levels of recording. Rather than pre and post comparisons, a more sophisticated analysis could be run using interrupted or other time series models, but we inspected pre-pandemic patterns and discerned no clear trends that needed adjustment.

We examined several patient subgroups, but future work could look at patterns by hospital trust and specialty. We saw falls in first and follow-up visits, and one could track whether clinic discharge rates changed during the period. In particular, it will be important to follow patients up to observe their future outcomes, although linkage with primary care records would be needed to capture patients who were not referred to OPDs.

Implications for policy and practice

It is reassuring that despite huge changes in clinical practice, face-to-face appointments for babies under 1 year were relatively preserved and that activity changes were similar among CYP living in more and less deprived areas. This suggests that the NHS continued to provide a responsive, equitable and service for CYP needing specialist care throughout the pandemic, albeit at a reduced level.

While part of the reduction in activity may be accounted for by reduced prevalence of infective conditions and greater parental empowerment and confidence in managing self-limiting illness (4,38), there is likely to be significant ongoing unmet need for specialist paediatric care. Whereas the impact of delay in accessing acute care can be assessed fairly quickly using routinely collected data, the impact of reduced access to outpatient services may be reflected more in worse quality of life for CYP and potentially worse longer term outcomes through lack of early diagnosis and early intervention (39). Identifying and addressing these unmet needs should be a priority for cilnicians, commissioners and policy makers.

For peer review only

These findings may also usefully inform the current debate about integrated care for CYP and the best ways of combining high quality care with convenience and positive experience for patients and families. In particular, greater use of remote care and patient family-initiated follow-up have huge potential benefits as well as potential risks in lower quality care or increasing inequalities. Further research is needed to understand and quantify these risks to guide mitigation strategies.

Conclusions

The impact on patients and the NHS of such a huge fall from pre-pandemic levels in OPD appointments, including those in surgical specialties, is yet to be felt. The extent to which the fall in total appointments and the rise in the use of telephones matters in terms of patient health outcomes will need further monitoring. The natural experiment of radical changes to paediatric outpatient care during the pandemic period may also inform development of more integrated and responsive specialist services for CYP in the future.

Acknowledgments

Contributorship statement

AB and DH devised the study. AB devised the approach to and conducted the data analysis. FN and AB drafted the manuscript. All authors contributed to the interpretation of the results and critical revision of the manuscript and approved the final draft.

Competing interests

None declared.

Funding

This study was funded by the National Institute for Health Research grant number NIHR202322 ‘Understanding the disruption of children and young people’s health and healthcare use during and after COVID-19 to inform healthcare and policy responses.’ The Dr Foster Unit is an academic unit in the Department of Primary Care and Public Health, within the School of Public Health, Imperial College London. The unit received research funding from Dr Foster Intelligence, an independent health service research organization (a wholly owned subsidiary of Telstra). The Dr Foster Unit at Imperial is affiliated with the National Institute of Health Research (NIHR) Imperial Patient Safety Translational Research Centre. The NIHR Imperial Patient Safety Translational Centre is a partnership between the Imperial College Healthcare NHS Trust and Imperial College London. The Department of Primary Care & Public Health at Imperial College London is grateful for support from the NW London NIHR Applied Research Collaboration and the Imperial NIHR Biomedical Research Centre. The views expressed in this publication are those of the authors and not necessarily those of the NIHR or the Department of Health and Social Care.

For peer review only

Data sharing statement

The pseudonymised patient data that were used for this study can be accessed by contacting NHS Digital (see https://digital.nhs.uk/services/data-access-request-service-dars). Access to these data is subject to a data sharing agreement (DSA) containing detailed terms and conditions of use following protocol approval from NHS Digital.

References

1. Isba R, Edge R, Jenner R, Broughton E, Francis N, Butler J. Where have all the children gone? Decreases in paediatric emergency department attendances at the start of the COVID-19 pandemic of 2020. Arch Dis Child. 2020 Jul 1;105(7):704–704.

2. Scaramuzza A, Tagliaferri F, Bonetti L, Soliani M, Morotti F, Bellone S, et al. Changing admission patterns in paediatric emergency departments during the COVID-19 pandemic. Arch Dis Child. 2020 Jul 1;105(7):704–6.

3. Rose K, Zyl KV, Cotton R, Wallace S, Cleugh F. Paediatric Attendances and Acuity in the Emergency Department during the COVID-19 Pandemic. medRxiv. 2020 Aug 11;2020.08.05.20168666.

4. Roland D, Harwood R, Bishop N, Hargreaves D, Patel S, Sinha I. Children's emergency presentations during the COVID-19 pandemic. Lancet Child Adolesc Health. 2020 Aug 1;4(8):e32–3.

5. McDonnell T, Nicholson E, Conlon C, Barrett M, Cummins F, Hensey C, et al. Assessing the Impact of COVID-19 Public Health Stages on Paediatric Emergency Attendance. Int J Environ Res Public Health [Internet]. 2020 Sep [cited 2021 May 6];17(18). Available from: https://www.ncbi.nlm.nih.gov/pmc/articles/PMC7558983/

6. Williams TC, MacRae C, Swann OV, Haseeb H, Cunningham S, Davies P, et al. Indirect effects of the COVID-19 pandemic on paediatric healthcare use and severe disease: a retrospective national cohort study. Arch Dis Child. 2021 Jan 15;

7. Gill PJ, Mahant S, Hall M, Berry JG. Reasons for Admissions to US Children's Hospitals During the COVID-19 Pandemic. JAMA. 2021 Apr 27;325(16):1676–9.

8. Dann L, Fitzsimons J, Gorman KM, Hourihane J, Okafor I. Disappearing act: COVID-19 and paediatric emergency department attendances. Arch Dis Child. 2020 Aug 1;105(8):810–1.

9. Wyatt S, Mohammed MA, Fisher E, McConkey R, Spilsbury P. Impact of the SARS-CoV-2 pandemic and associated lockdown measures on attendances at emergency departments in English hospitals: A retrospective database study. Lancet Reg Health – Eur [Internet]. 2021 Mar 1 [cited 2021 May 7];2. Available from: https://www.thelancet.com/journals/lanepe/article/PIIS2666-7762(21)00011-9/abstract

For peer review only

10. Creese H, Taylor-Robinson D, Saglani S, Saxena S. Primary care of children and young people with asthma during the COVID-19 era. Br J Gen Pract. 2020 Nov 1;70(700):528–9.

11. Sheath C, Abdelrahman M, MacCormick A, Chan D. Paediatric appendicitis during the COVID-19 pandemic. J Paediatr Child Health. 2021 Feb 1;

12. Gajaweera H, Oladele D, Connett G. Evaluation of the impact of shielding to avoid COVID-19 infection on respiratory symptoms in children with severe asthma. Arch Dis Child. 2021 Apr;106(4):e23.

13. Research Network PST, Arthur F, Harwood R, Allin B, Bethell GS, Boam T, et al. Lockdown during the COVID-19 pandemic: impact on infants with pyloric stenosis. Arch Dis Child. 2020 Dec 1;

14. Diabetes C-19 PUIG of CC and, Williams G, McLean R, Liu J-F, Ritzmann T, Dandapani M, et al. A multi-centre service evaluation of the impact of the COVID-19 pandemic on presentation of newly diagnosed cancers and type 1 diabetes in children in the UK. medRxiv. 2021 Feb 21;2021.02.09.21251149.

15. Langan D, Shelmerdine S, Taylor A, Bryant WA, Booth J, Sebire NJ, et al. Impact of the COVID-19 pandemic on radiology appointments in a tertiary children's hospital: a retrospective study. BMJ Paediatr Open. 2021 Aug 1;5(1):e001210.

16. British Medical Association. The hidden impact of COVID-19 on patient care in the NHS in England [Internet]. 2020 Jul. Available from: https://www.bma.org.uk/media/2841/the-hidden-impact-of-covid_web-pdf.pdf

17. Thorlby R, Fraser C, Gardner T. Non-COVID-19 NHS care during the pandemic | The Health Foundation [Internet]. [cited 2021 May 7]. Available from: https://www.health.org.uk/news-and-comment/charts-and-infographics/non-covid-19-nhs-care-during-the-pandemic

18. Ruzangi J, Blair M, Cecil E, Greenfield G, Bottle A, Hargreaves DS, et al. Trends in healthcare use in children aged less than 15 years: a population-based cohort study in England from 2007 to 2017. BMJ Open. 2020 May 1;10(5):e033761.

19. Galen LS van, Car J. Telephone consultations. BMJ. 2018 Mar 29;360:k1047.

20. Vaona A, Pappas Y, Grewal RS, Ajaz M, Majeed A, Car J. Training interventions for improving telephone consultation skills in clinicians. Cochrane Database Syst Rev. 2017 Jan 5;1:CD010034.

21. Armstrong S. Universal access to the digital NHS—but only if you have a smartphone. BMJ. 2021 Jul 15;374:n1732.

22. Jessiman PE, Powell K, Williams P, Fairbrother H, Crowder M, Williams JG, et al. A systems map of the determinants of child health inequalities in England at the local level. PloS One. 2021 Feb 12;16(2):e0245577–e0245577.

For peer review only

23. Elbarbary NS, Dos Santos TJ, de Beaufort C, Agwu JC, Calliari LE, Scaramuzza AE. COVID-19 outbreak and pediatric diabetes: Perceptions of health care professionals worldwide. Pediatr Diabetes. 2020 Nov;21(7):1083–92.

24. Ingram M-CE, Raval MV, Newton C, Lopez ME, Berman L. Characterization of initial North American pediatric surgical response to the COVID-19 pandemic. J Pediatr Surg. 2020 Aug 1;55(8):1431–5.

25. Wirrell EC, Grinspan ZM, Knupp KG, Jiang Y, Hammeed B, Mytinger JR, et al. Care Delivery for Children With Epilepsy During the COVID-19 Pandemic: An International Survey of Clinicians. J Child Neurol. 2020 Nov;35(13):924–33.

26. Sugand K, Park C, Morgan C, Dyke R, Aframian A, Hulme A, et al. Impact of the COVID-19 pandemic on paediatric orthopaedic trauma workload in central London: a multi-centre longitudinal observational study over the 'golden weeks'. Acta Orthop. 2020 Dec;91(6):633–8.

27. Duncan H, Russell RK. Role for structured telephone clinics in paediatric gastroenterology: reflections, lessons and patient feedback. BMJ Open Gastroenterol. 2019;6(1):e000245.

28. O'Brien G, Lazebnik R. Telephone call reminders and attendance in an adolescent clinic. Pediatrics. 1998 Jun;101(6):E6.

29. Leibar Tamayo A, Linares Espinós E, Ríos González E, Trelles Guzmán C, Álvarez-Maestro M, de Castro Guerín C, et al. Evaluation of teleconsultation system in the urological patient during the COVID-19 pandemic. Actas Urol Esp. 2020 Nov;44(9):617–22.

30. Darr A, Senior A, Argyriou K, Limbrick J, Nie H, Kantczak A, et al. The impact of the coronavirus (COVID-19) pandemic on elective paediatric otolaryngology outpatient services - An analysis of virtual outpatient clinics in a tertiary referral centre using the modified paediatric otolaryngology telemedicine satisfaction survey (POTSS). Int J Pediatr Otorhinolaryngol. 2020 Nov;138:110383.

31. Wolthers TO, Wolthers OD. Telephone consultation as a substitute for face-to-face consultation during the COVID-19 pandemic. Dan Med J. 2020 Jun 23;67(7).

32. Rametta SC, Fridinger SE, Gonzalez AK, Xian J, Galer PD, Kaufman M, et al. Analyzing 2,589 child neurology telehealth encounters necessitated by the COVID-19 pandemic. Neurology. 2020 Sep 1;95(9):e1257–66.

33. Shaw S, Wherton J, Vijayaraghavan S, Morris J, Bhattacharya S, Hanson P, et al. Advantages and limitations of virtual online consultations in a NHS acute trust: the VOCAL mixed-methods study. Health Serv Deliv Res. 2018 Jun 22;6(21):162.

34. Badawy SM, Radovic A. Digital Approaches to Remote Pediatric Health Care Delivery During the COVID-19 Pandemic: Existing Evidence and a Call for Further Research. JMIR Pediatr Parent. 2020 Jun 25;3(1):e20049–e20049.

For peer review only

35. Charnell AM, Hoen L ’t, Sforza S, Spinoit A-F, Radford A. Remote consultations in paediatric urology-Not just for pandemics? J Pediatr Urol. 2021 Apr;17(2):260–2.

36. Cohen E, Cohen MI. COVID-19 will forever change the landscape of telemedicine. Curr Opin Cardiol. 2021 Jan;36(1):110–5.

37. Chen Y, Kathirithamby DR, Li J, Candelario-Velazquez C, Bloomfield A, Ambrose AF. Telemedicine in the Coronavirus Disease 2019 Pandemic: A Pediatric Rehabilitation Perspective. Am J Phys Med Rehabil. 2021 Apr;100(4):321–6.

38. Chang AY, Cullen MR, Harrington RA, Barry M. The impact of novel coronavirus COVID-19 on noncommunicable disease patients and health systems: a review. J Intern Med. 2020/10/27 ed. 2021 Apr;289(4):450–62.

39. Hargreaves DS, Elliott MN, Viner RM, Richmond TK, Schuster MA. Unmet Health Care Need in US Adolescents and Adult Health Outcomes. Pediatrics. 2015 Sep 1;136(3):513–20.

For peer review only

Figure Legends

Figure 1. All appointments since Jan 2018 by age group

Figure 2. Trends in total appointments by IMD fifth for ages 0-1 and 20-24; IMD 1 is least deprived, IMD 5 is most deprived

For peer review only

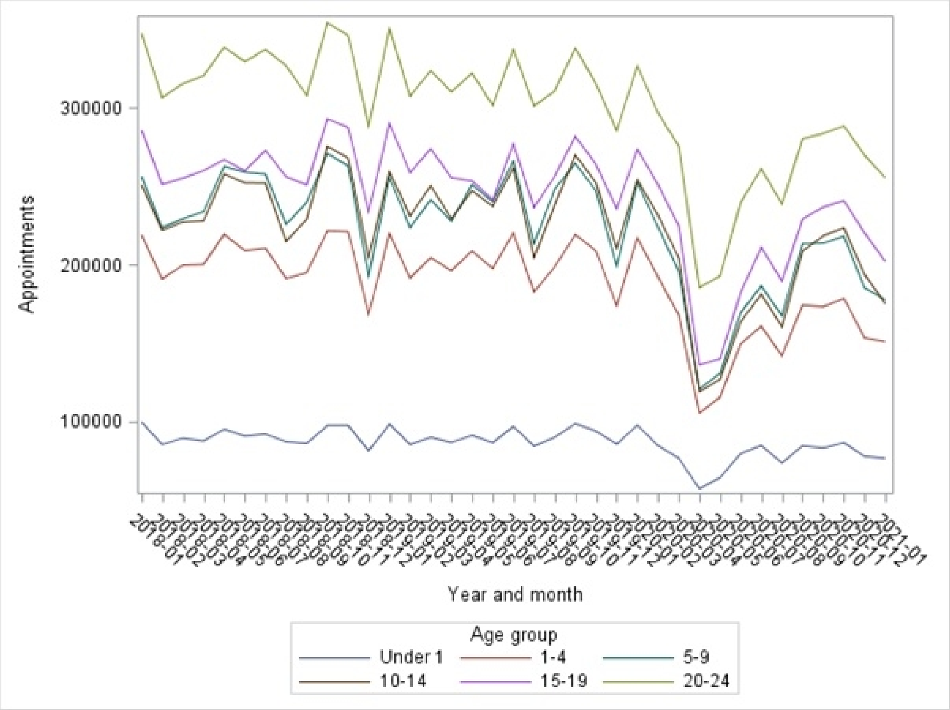

Figure 1. All scheduled appointments since Jan 2018 by age group

53x40mm (300 x 300 DPI)

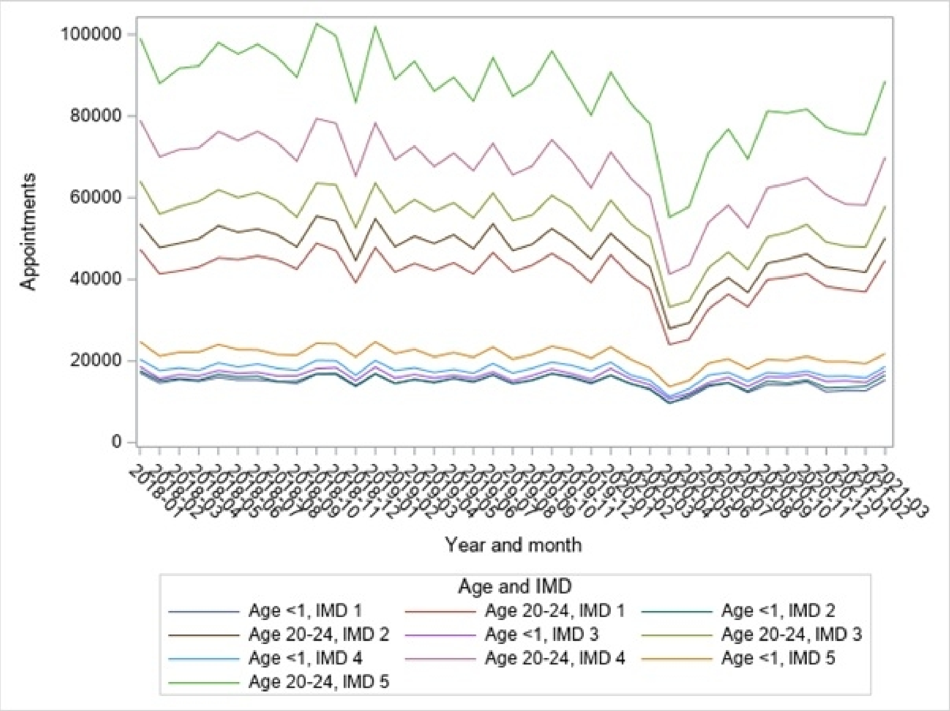

Figure 2. Trends in total appointments by IMD fifth for ages 0-1 and 20-24; IMD 1 is least deprived, IMD 5 is most deprived

53x40mm (300 x 300 DPI)

Supplementary material

Figure S1. All appointments since Jan 2018 by sex for three age groups

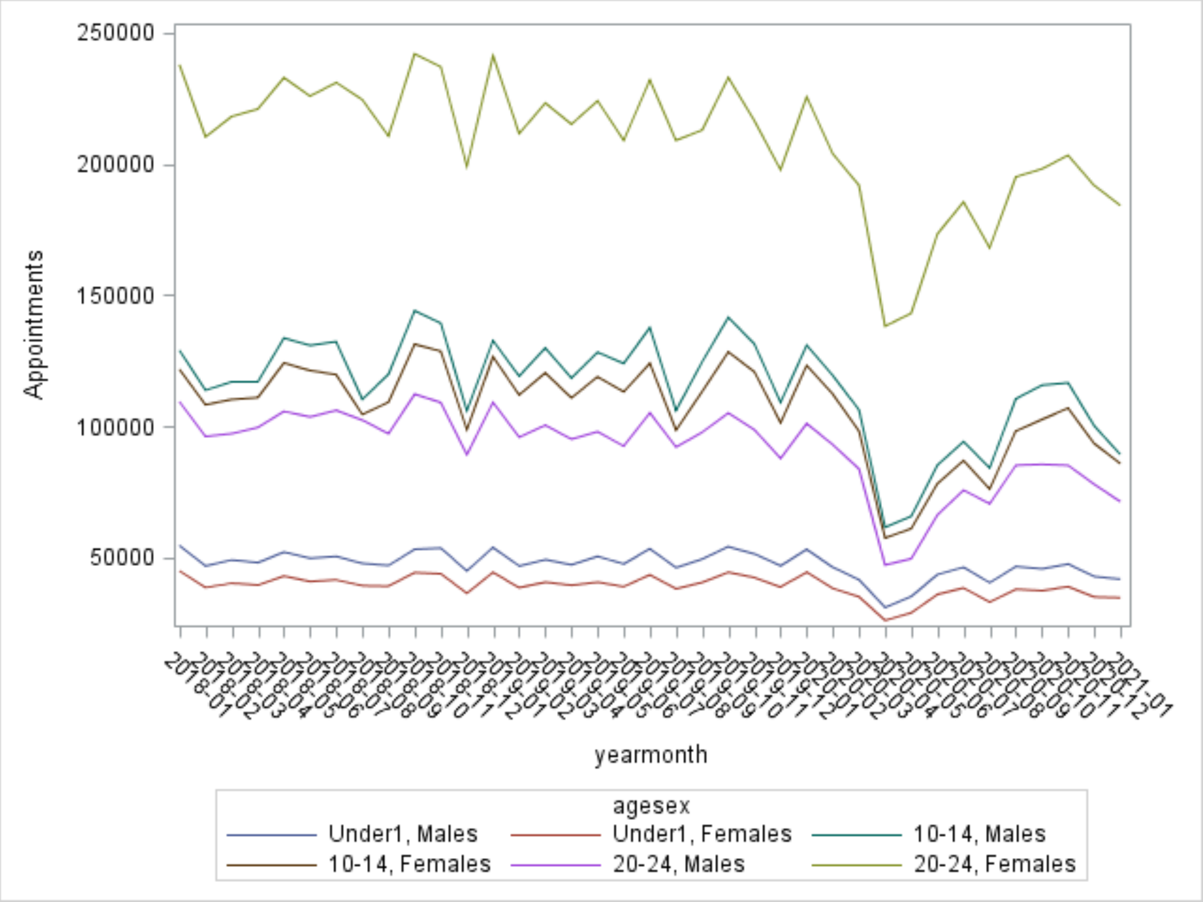

For peer review only

Figure S2. All appointments, all ages, split by whether the specialty was surgical

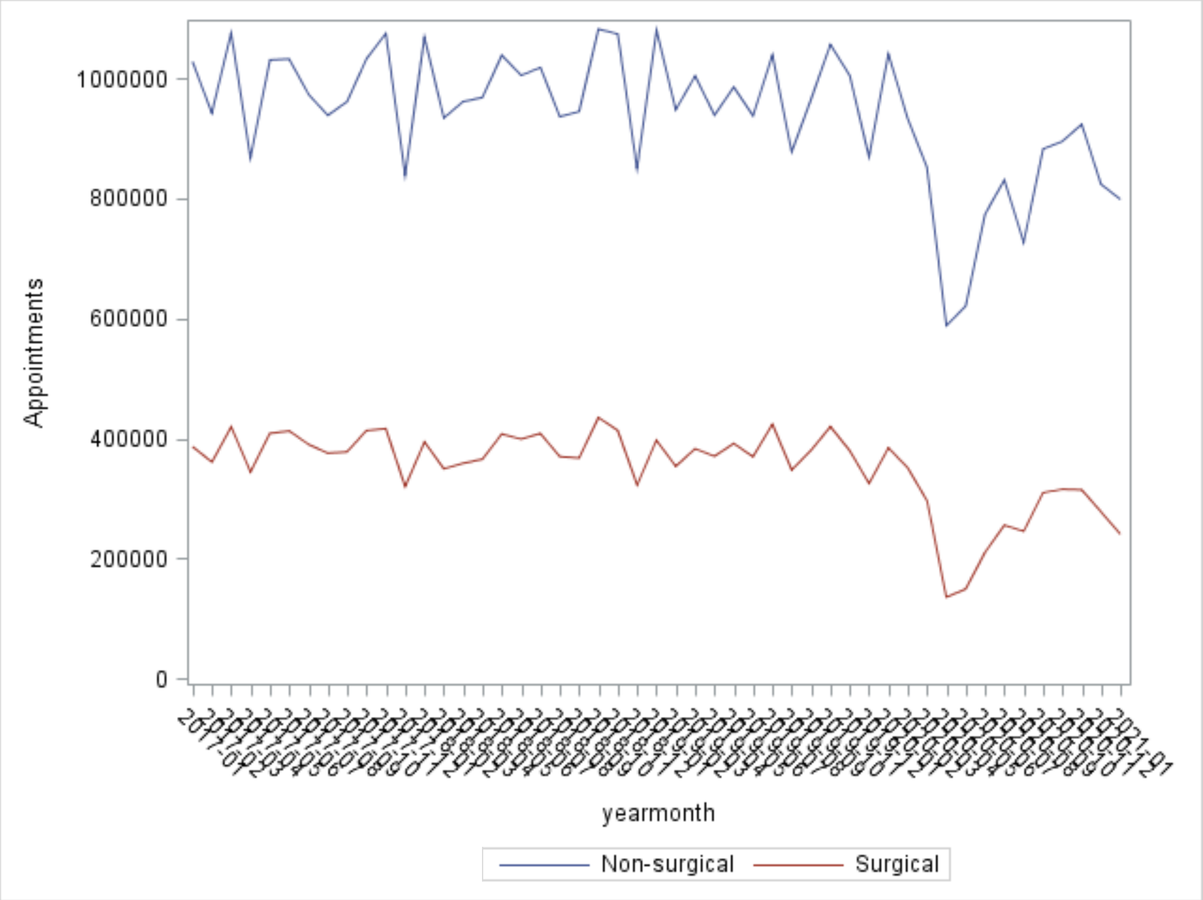

Figure S3. Trends in first appointments (both face-to-face and telephone) by IMD fifth; IMD 1 is least deprived, IMD 5 most deprived

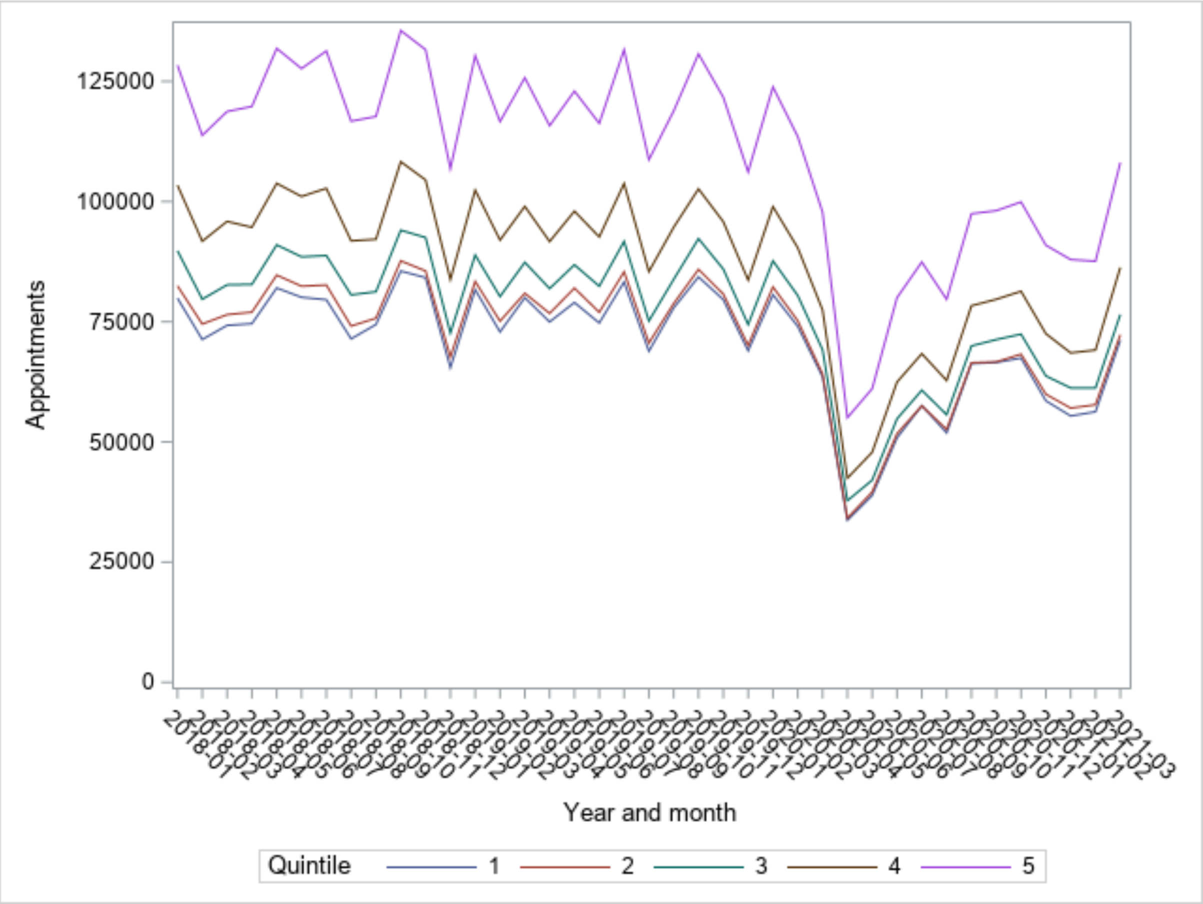

Figure S4. Trends in follow up appointments (both face-to-face and telephone) by IMD fifth; IMD 1 is least deprived, IMD 5 most deprived

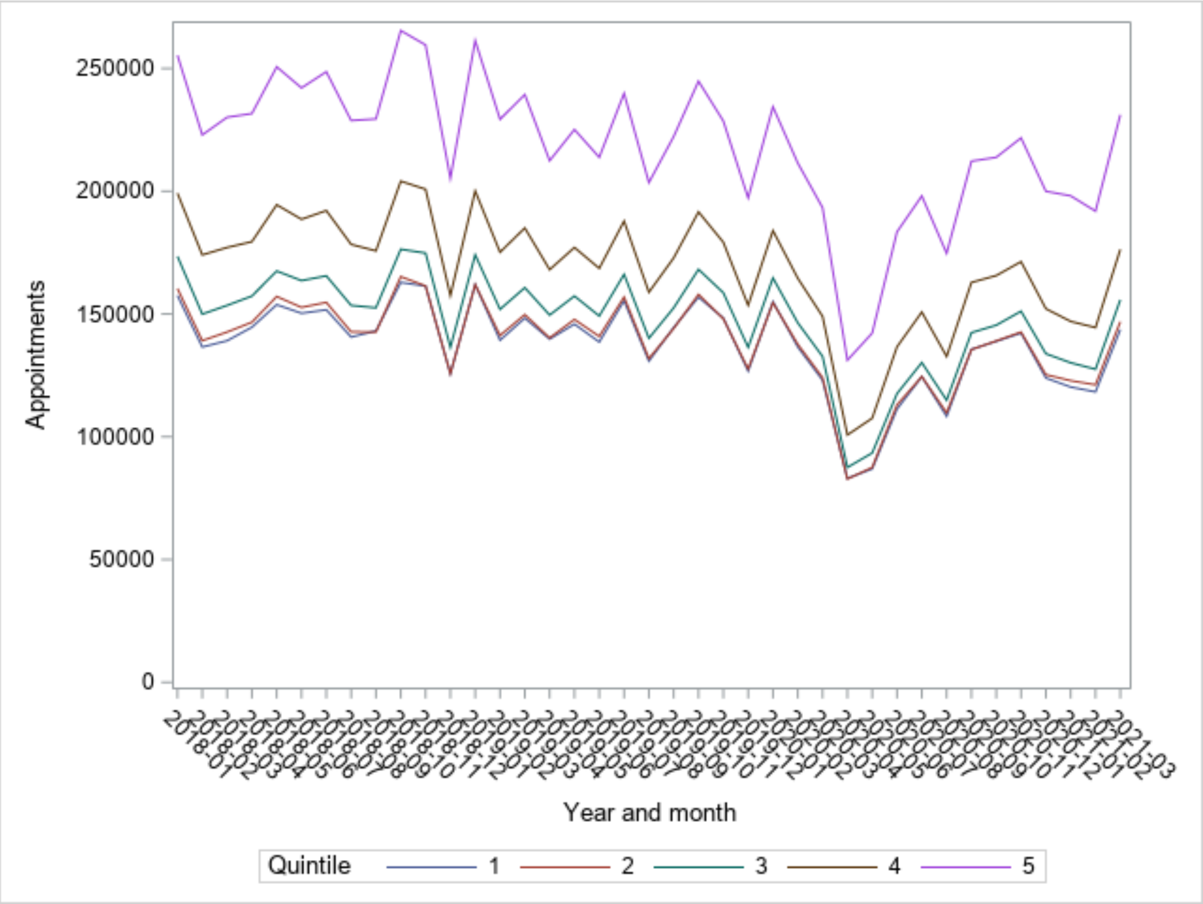

Figure S5. Increase compared with three-year average for 1st phone appointments

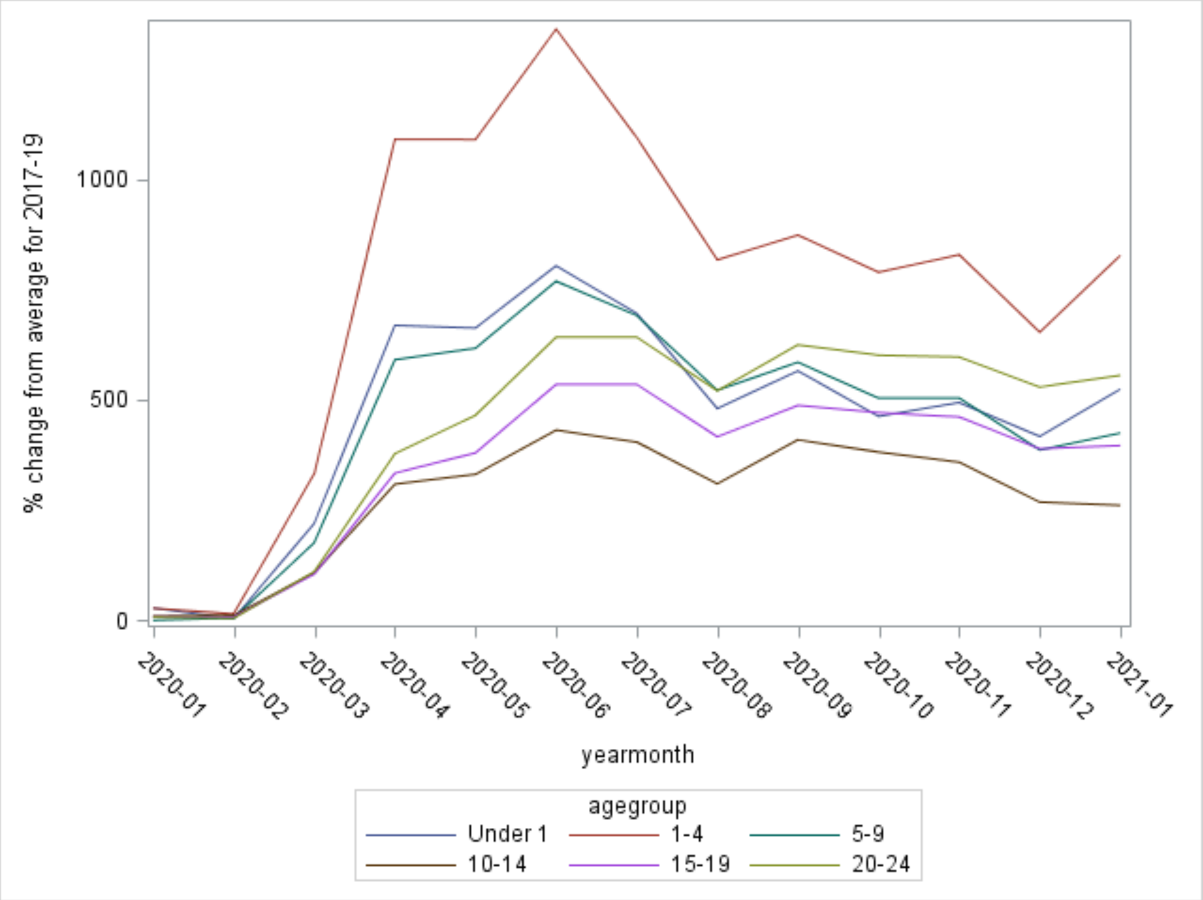

For peer review only

Figure S6. Attendance rates for the under-1s, comparing F2F with phone appts

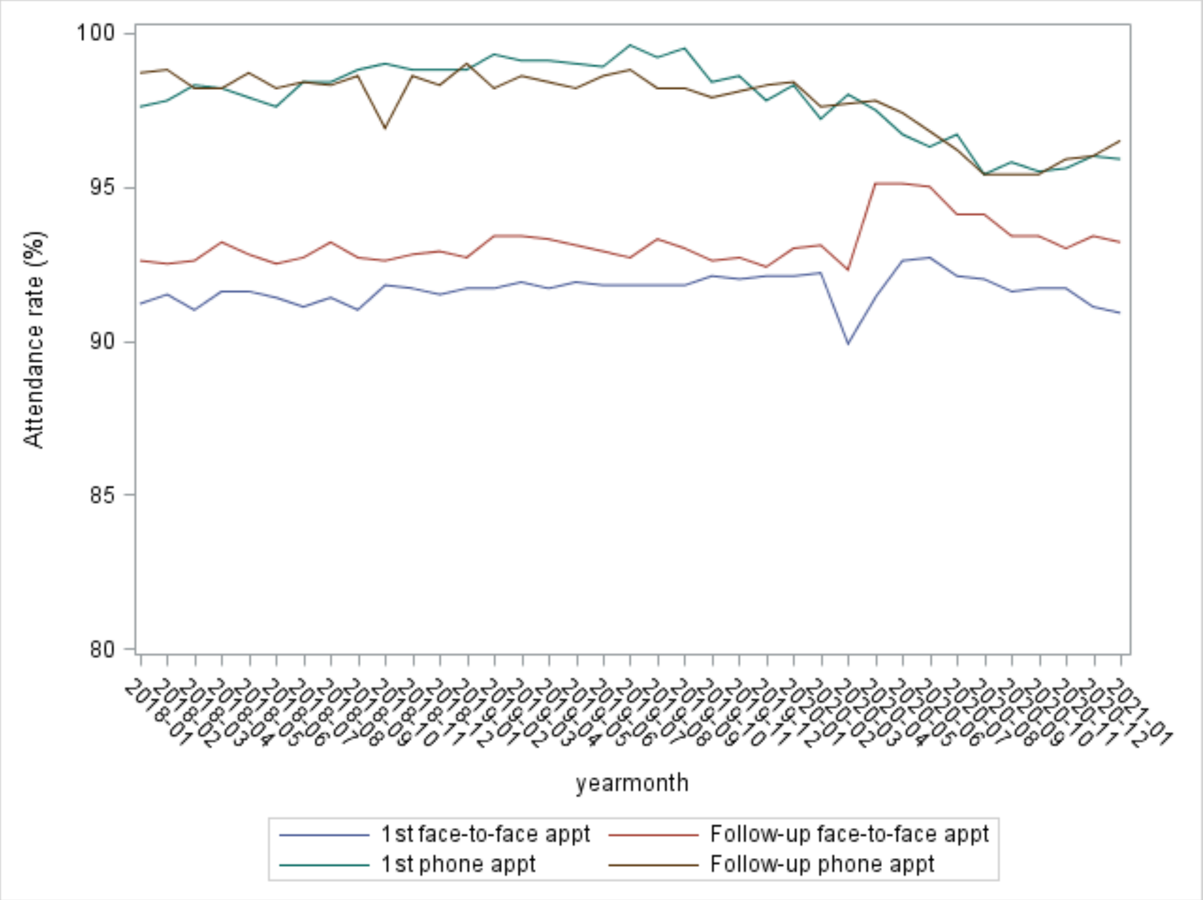

The pattern is repeated for the other age groups. For example, here are the attendance rates for those aged 20-24:

Figure S7. Attendance rates for the 20-24s, comparing F2F with phone appts

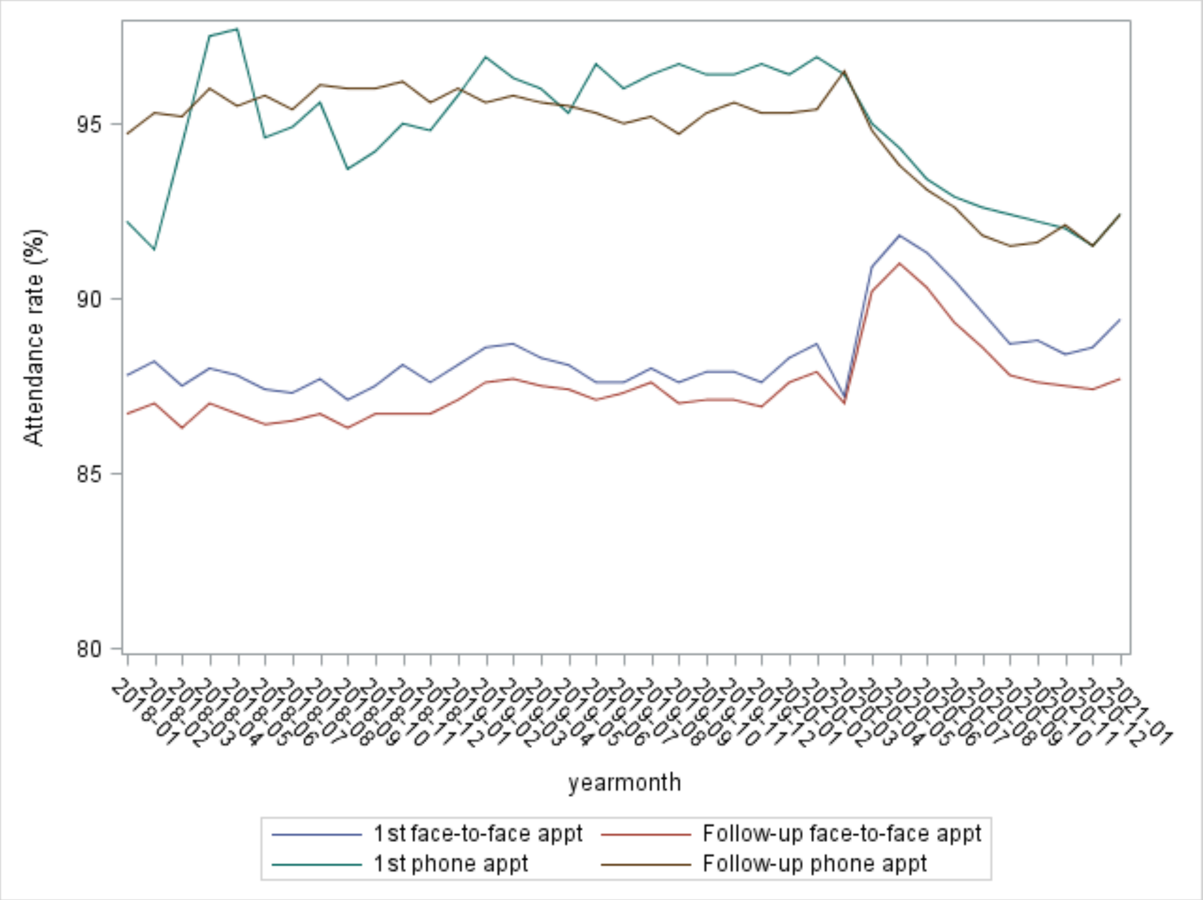

For peer review only

STROBE 2007 (v4) checklist of items to be included in reports of observational studies in epidemiology*
Checklist for cohort, case-control, and cross-sectional studies (combined)

Section/Topic	Item #	Recommendation	Reported on page #
**Title and abstract**	1	(*a*) Indicate the study's design with a commonly used term in the title or the abstract	1
		(*b*) Provide in the abstract an informative and balanced summary of what was done and what was found	1
**Introduction**			
Background/rationale	2	Explain the scientific background and rationale for the investigation being reported	2
Objectives	3	State specific objectives, including any pre-specified hypotheses	2
**Methods**			
Study design	4	Present key elements of study design early in the paper	3
Setting	5	Describe the setting, locations, and relevant dates, including periods of recruitment, exposure, follow-up, and data collection	3
Participants	6	(*a*) *Cohort study*—Give the eligibility criteria, and the sources and methods of selection of participants. Describe methods of follow-up<br>*Case-control study*—Give the eligibility criteria, and the sources and methods of case ascertainment and control selection. Give the rationale for the choice of cases and controls<br>*Cross-sectional study*—Give the eligibility criteria, and the sources and methods of selection of participants	3
		(*b*) *Cohort study*—For matched studies, give matching criteria and number of exposed and unexposed<br>*Case-control study*—For matched studies, give matching criteria and the number of controls per case	
Variables	7	Clearly define all outcomes, exposures, predictors, potential confounders, and effect modifiers. Give diagnostic criteria, if applicable	3
Data sources/ measurement	8*	For each variable of interest, give sources of data and details of methods of assessment (measurement). Describe comparability of assessment methods if there is more than one group	*3*
Bias	9	Describe any efforts to address potential sources of bias	3
Study size	10	Explain how the study size was arrived at	3
Quantitative variables	11	Explain how quantitative variables were handled in the analyses. If applicable, describe which groupings were chosen and why	3
Statistical methods	12	(*a*) Describe all statistical methods, including those used to control for confounding	3
		(*b*) Describe any methods used to examine subgroups and interactions	3
		(*c*) Explain how missing data were addressed	3
		(*d*) *Cohort study*—If applicable, explain how loss to follow-up was addressed<br>*Case-control study*—If applicable, explain how matching of cases and controls was addressed	N/A

For peer review only

		Cross-sectional study—If applicable, describe analytical methods taking account of sampling strategy	
		(*e*) Describe any sensitivity analyses	N/A
**Results**			
Participants	13*	(a) Report numbers of individuals at each stage of study—eg numbers potentially eligible, examined for eligibility, confirmed eligible, included in the study, completing follow-up, and analysed	3
		(b) Give reasons for non-participation at each stage	N/A
		(c) Consider use of a flow diagram	N/A
Descriptive data	14*	(a) Give characteristics of study participants (eg demographic, clinical, social) and information on exposures and potential confounders	4
		(b) Indicate number of participants with missing data for each variable of interest	N/A
		(c) *Cohort study*—Summarise follow-up time (eg, average and total amount)	4
Outcome data	15*	*Cohort study*—Report numbers of outcome events or summary measures over time	
		Case-control study—Report numbers in each exposure category, or summary measures of exposure	
		Cross-sectional study—Report numbers of outcome events or summary measures	4
Main results	16	(*a*) Give unadjusted estimates and, if applicable, confounder-adjusted estimates and their precision (eg, 95% confidence interval). Make clear which confounders were adjusted for and why they were included	N/A
		(*b*) Report category boundaries when continuous variables were categorized	4
		(*c*) If relevant, consider translating estimates of relative risk into absolute risk for a meaningful time period	N/A
Other analyses	17	Report other analyses done—eg analyses of subgroups and interactions, and sensitivity analyses	4
**Discussion**			
Key results	18	Summarise key results with reference to study objectives	4-5
Limitations	19	Discuss limitations of the study, taking into account sources of potential bias or imprecision. Discuss both direction and magnitude of any potential bias	6
Interpretation	20	Give a cautious overall interpretation of results considering objectives, limitations, multiplicity of analyses, results from similar studies, and other relevant evidence	6-7
Generalisability	21	Discuss the generalisability (external validity) of the study results	6-7
**Other information**			
Funding	22	Give the source of funding and the role of the funders for the present study and, if applicable, for the original study on which the present article is based	7

*Give information separately for cases and controls in case-control studies and, if applicable, for exposed and unexposed groups in cohort and cross-sectional studies.
Note: An Explanation and Elaboration article discusses each checklist item and gives methodological background and published examples of transparent reporting. The STROBE checklist is best used in conjunction with this article (freely available on the Web sites of PLoS Medicine at http://www.plosmedicine.org/, Annals of Internal Medicine at http://www.annals.org/, and Epidemiology at http://www.epidem.com/). Information on the STROBE Initiative is available at www.strobe-statement.org.

For peer review only

BMJ Open

Impact of Covid-19 on outpatient appointments in children and young people in England: an observational study

Journal:	*BMJ Open*
Manuscript ID	bmjopen-2022-060961.R1
Article Type:	Original research
Date Submitted by the Author:	11-May-2022
Complete List of Authors:	Bottle, Alex; Imperial College London, Department of Primary Care and Public Health<br>Neale, Francesca; Imperial College London, Department of Primary Care and Public Health<br>Foley, Kimberley; Imperial College London, Department of Primary Care and Public Health<br>Viner, Russell; UCL Great Ormond Street Institute of Child Health Population Policy and Practice<br>Kenny, Simon; NHS England and NHS Improvement London; University of Liverpool, Institute of Systems, Molecular and Integrative Biology<br>Aylin, Paul; Imperial College London, Department of Primary Care and Public Health<br>Saxena, Sonia; Imperial College London, Department of Primary Care and Public Health<br>Hargreaves, Dougal; Imperial College London, Department of Primary Care and Public Health
**Primary Subject Heading**:	Paediatrics
Secondary Subject Heading:	Public health
Keywords:	PAEDIATRICS, COVID-19, PUBLIC HEALTH

SCHOLARONE™
Manuscripts

BMJ

I, the Submitting Author has the right to grant and does grant on behalf of all authors of the Work (as defined in the below author licence), an exclusive licence and/or a non-exclusive licence for contributions from authors who are: i) UK Crown employees; ii) where BMJ has agreed a CC-BY licence shall apply, and/or iii) in accordance with the terms applicable for US Federal Government officers or employees acting as part of their official duties; on a worldwide, perpetual, irrevocable, royalty-free basis to BMJ Publishing Group Ltd ("BMJ") its licensees and where the relevant Journal is co-owned by BMJ to the co-owners of the Journal, to publish the Work in this journal and any other BMJ products and to exploit all rights, as set out in our licence.

The Submitting Author accepts and understands that any supply made under these terms is made by BMJ to the Submitting Author unless you are acting as an employee on behalf of your employer or a postgraduate student of an affiliated institution which is paying any applicable article publishing charge ("APC") for Open Access articles. Where the Submitting Author wishes to make the Work available on an Open Access basis (and intends to pay the relevant APC), the terms of reuse of such Open Access shall be governed by a Creative Commons licence – details of these licences and which Creative Commons licence will apply to this Work are set out in our licence referred to above.

Other than as permitted in any relevant BMJ Author's Self Archiving Policies, I confirm this Work has not been accepted for publication elsewhere, is not being considered for publication elsewhere and does not duplicate material already published. I confirm all authors consent to publication of this Work and authorise the granting of this licence.

For peer review only

Impact of COVID-19 on outpatient appointments in children and young people in England: an observational study

Alex Bottle[1], Francesca K Neale[1], Kimberley A Foley[1], Russel Viner[2], Simon E Kenny[3,4,5], Paul Aylin[1], Sonia Saxena[1], Dougal S Hargreaves[1]

1. Department of Primary Care and Public Health, Imperial College London School of Public Health, London, UK
2. Population, Policy and Practice Research Programme, UCL Institute of Child Health, London, UK
3. NHS England and NHS Improvement, London, UK
4. Institute of Systems, Molecular and Integrative Biology, University of Liverpool, Liverpool, UK
5. Department of Paediatric Surgery, Alder Hey in the Park, Liverpool, UK

Correspondence to:
Dr Francesca Neale, Department of Primary Care and Public Health, Imperial College London School of Public Health, London, W6 8RP, UK; 020 7594 3368; francesca.neale1@nhs.net

Keywords:
Outpatient appointments
Children
Young people
Health service use
Covid-19

Word count: 2968 (excluding title page, references, figures and tables)

For peer review only

Impact of COVID-19 on outpatient appointments in children and young people in England: an observational study

Abstract

Objectives: To describe the impact of the Covid-19 pandemic on outpatient appointments for children and young people.

Setting: All National Health Service (public) hospitals in England.

Participants: All people in England aged <25 years.

Outcome measures: Outpatient department attendance numbers, rates and modes (face-to-face vs telephone) by age group, sex, and socio-economic deprivation.

Results: Compared with the average for January 2017 to December 2019, there was a 3.8 million appointment shortfall (23.5%) for the under-25 population in England between March 2020 and February 2021, despite a total rise in phone appointments of 2.6 million during that time. This was true for each age group, sex and deprivation fifth, but there were smaller decreases in face-to-face and total appointments for babies under 1 year. For all ages combined, around one in six first and one in four follow-up appointments were by phone in the most recent period. The proportion of appointments attended was high, at over 95% for telephone and over 90% for face-to-face appointments for all ages.

Conclusions: Covid-19 led to a dramatic fall in total outpatient appointments and a large rise in the proportion of those appointments conducted by telephone. The impact that this has had on patient outcomes is still unknown. The differential impact of Covid-19 on outpatient

For peer review only

activity in different socio-demographic groups may also inform design of paediatric outpatient services in the post-Covid period.

Strengths and Limitations

- This paper benefits from national data collected in a standardised way.
- The data quality for administrative items like dates, age and sex is very high.
- We were unable to analyse patterns by diagnosis due to very low levels of recording.
- Although we examined several patient subgroups, future work could look at patterns by hospital trust and specialty.

For peer review only

Introduction

The Covid-19 pandemic has led to profound disruption to health services globally as treatment of Covid-19 infections was prioritised over some groups felt to be at lower risk, including children and young people. In the United Kingdom (UK), strict lockdown measures were introduced in March 2020, November 2020 and January 2021. The disruptions to daily life and health services, including the temporary closure of some paediatric wards and emergency departments, led to changes to health seeking behaviour and health service use. Reductions in emergency department attendance and emergency admissions have been documented in both children (1–8) and adults (9). A national retrospective cohort study in Scotland described reductions of almost two-thirds in paediatric unscheduled primary care presentations and half of emergency secondary care presentations, without any change in Paediatric Intensive Care Unit admissions or mortality (6). Falls in urgent care contacts compared with previous years led to speculation that children and young people's (CYP) health might deteriorate if serious conditions were missed or if they experienced delays in diagnosis of chronic disease. However, studies have found no significant impact on route to diagnosis and severity of presentation for type 1 diabetes and new diagnosed cancers, as well as no impact on overall outcomes for asthma, pyloric stenosis and appendicitis (10–14).

There have also been concerns that some CYP were unable to access specialist services in hospitals. For example, a study of radiology outpatient activity at a tertiary children's hospital from March 2020-March 2021 showed a reduction of 27.3% (15). The British Medical Association estimated that across all ages in England between April and June 2020 there were between 2.47 million and 2.6 million fewer first outpatient attendances than expected (16). Similarly, The Health Foundation reported a fall of 4.4 million outpatient

For peer review only - http://bmjopen.bmj.com/site/about/guidelines.xhtml

appointments in England in May 2020 compared with May 2019 and 4 million fewer GP referrals to outpatient appointments between January and October 2020 compared with the same period in 2019, although referrals for suspected cancer had recovered to pre-pandemic levels by October 2020 (17). However, no detailed analysis has been published of paediatric outpatient activity, where growing concerns had already been raised about the equity of access to specialist care pre-Covid (18).

Telephone and video consultations have been used prior to the Covid-19 pandemic in many specialties, including paediatrics, with advantages including less time wasted between appointments, no associated costs, fewer late arrivals and, since the Covid-19 pandemic, patients are understandably anxious about the risk of catching Covid-19 in hospital. Disadvantages of remote consultations include missing non-verbal cues, the exclusion of those without access to technology, the inability to examine the patient, and that many doctors have not been trained to conduct telephone consultations (19–21). However, it is unknown to what extent remote paediatric consultations were used during the Covid-19 pandemic.

We make use of England's national hospital administrative database, Hospital Episodes Statistics (HES), to describe trends in outpatient appointment numbers, rates and modes (face-to-face vs telephone) in CYP by age group, sex, and socio-economic deprivation between January 2017 and January 2021 in England.

Methods

Data

We extracted HES data for outpatient appointments between 1st Jan 2017 and 28th Feb 2021 and for patients aged under 25. Records were excluded if either the patient or the hospital cancelled the appointment (between 7.7% and 10.4% of appointments in 2019-20, for instance, depending on the age group) or in the rare cases when the age or sex was invalid. Area-level Index of Multiple Deprivation (IMD) was attached to HES for each patient and turned into population-weighted fifths so that nationally, for all ages combined, each fifth contained equal population. The ATENTYPE field distinguishes between first and follow-up appointments, with separate values for face-to-face, telephone and not specified; we excluded records for 'not specified' (0.2%-0.6% of appointments for the whole period, depending on the age group) when comparing face-to-face and telephone. It also states whether the patient attended. The term telephone consultations refers to both telephone and video consultations as no separate value for video consultations is available.
The pre-pandemic period was defined as 1st Jan 2017 to 29th Feb 2020; the pandemic period was defined as 1st March 2020 onwards.

Statistical analysis

Appointment counts and attendance rates (i.e. proportion of appointments that the patient attended) were plotted by month and patient subgroup, including by age (under-1, 1-4, 5-9, 10-14, 15-19 and 20-24). For ease of comparison, changes in appointment numbers were calculated for the 12-month period from 1st Mar 2020 to 28th Feb 2021 rather than to the

For peer review only - http://bmjopen.bmj.com/site/about/guidelines.xhtml

end of Mar 2021. This was done by comparing total counts with age-matched yearly averages across the three-year period 1st Jan 2017 to 31st Dec 2019 rather than use time-series predictions, for example, as no clear monthly trends were noted during that time. Changes were expressed in absolute terms and as percentages. Due to the descriptive rather than hypothesis-driven nature of our study and the very large numbers involved, no statistical tests were performed.

Ethics

We had approval from the Secretary of State and the Health Research Authority under Regulation 5 of the Health Service (Control of Patient Information) Regulations 2002 to hold confidential data and analyse them for research purposes (CAG ref 15/CAG/0005). We have approval to use them for research and measuring quality of delivery of healthcare, from the London - South East Ethics Committee (REC ref 20/LO/0611).

Patient and Public Involvement

There was no patient or public involvement in this research.

Results

For CYP aged under 25 years, the overall fall in outpatient appointments for the 12-month period Mar 2020 to Feb 2021 compared with the means for the previous three years was over 3.8 million, a 23.5% fall, with the biggest percentage fall in the 5-9s and the smallest percentage change in under 1s (Table 1). This was despite a total rise in phone appointments of 2.6 million.

Table 1. Changes in numbers of scheduled appointments by age group for Mar 2020 to Feb 2021 compared with yearly average across the period Jan 2017 to Dec 2019

Age (years)	First F2F appt	Follow-up F2F appt	First phone appt	Follow-up phone appt	Total (% change)
Under 1	-167,106 (-28.1)	-124,745 (-27.1)	+50,189 (+542.4)	+69,267 (+311.0)	-172,395 (-15.9%)
1-4	-408,050 (-46.6)	-635,432 (-42.6)	+104,189 (+881.6)	+318,574 (+675.2)	-620,819 (-25.6%)
5-9	-424,378 (-47.5)	-819,892 (-43.1)	+103,778 (+514.9)	+395,336 (+639.2)	-745,056 (-25.9%)
10-14	-403,763 (-46.8)	-786,708 (-41.8)	+97,933 (+317.5)	+384,810 (+585.7)	-707,728 (-24.9%)
15-19	-388,535 (-42.4)	-835,778 (-39.5)	+116,457 (+412.0)	+383,934 (+559.8)	-723,922 (-23.1%)
20-24	-497,072 (-37.4)	-879,924 (-36.2)	+159,949 (+523.9)	+378,020 (+540.5)	-839,027 (-21.7%)
Total	-2,288,804 (-41.8)	-4,082,479 (-39.7)	+632,395 (+483.3)	+1,929,941 (+575.2)	-3,808,947 (-23.5%)

F2F = Face-to-face; "first" and "follow-up" are defined by HES

For peer review only - http://bmjopen.bmj.com/site/about/guidelines.xhtml

Total outpatient appointment numbers increased from May 2020 but had not reached pre-pandemic levels by Mar 2021 (Figure 1). This was true for each age group and sex (Appendix Figure S1) and for surgical and non-surgical specialties (Appendix Figure S2). The same pattern was observed for each IMD fifth (Appendix Figure S3, S4), which was unchanged since Mar 2020. To illustrate, Figure 2 shows this for the under-1s and those aged 20-24: for all age groups, the less-affluent areas had the most appointments.

Phone appointments increased by 15% from Mar 2020 to Feb 2021 for both first and follow-up visits. Table 2 compares pre-pandemic (Jan 2017 to Dec 2019) averages with Oct 2020 to Feb 2021 averages. The largest relative changes were seen among under 1s with tripling of the proportion of first appointments performed by phone (1.5% to 4.6%) and almost doubling of the proportion of follow-up appointments performed by phone (10.5% to 20.0%). The largest percentage rises were for the 10-14s (Table 2).

Table 2. Percentage of first and follow-up appointments that are by telephone, stratified by age and period (raw numbers are included in Appendix Table S1)

	Jan 2017 to Dec 2019 average		Oct 2020 to Feb 2021 average*	
Age (years)	First appt % by phone	Follow-up appt % by phone	First appt % by phone	Follow-up appt % by phone
Under-1	1.5	10.5	4.6	20.0
1-4	1.3	16.5	3.1	26.3
5-9	2.2	17.2	3.1	25.8
10-14	3.5	18.8	3.4	26.0
15-19	3.0	19.8	3.1	23.8
20-24	2.2	18.5	2.8	22.6
Total	2.3	17.3	3.2	24.5

* Oct 2020 to Feb 2021 is given because the first wave of covid-19 had ended and the system had stabilised

For all ages combined, around one in six first and one in four follow-up appointments were by phone in the most recent period. Appendix Figure S5 shows the rise in phone appointments by age relative to the pre-pandemic period.

Proportions of appointments attended

For peer review only

Attendance was high, at over 95% for telephone and over 90% for face-to-face appointments for all ages. There were slight falls in the former and slight rises in the latter from March 2021 (Appendix Figure S6, S7). Attendance and patterns did not differ by sex, with the exception that in the 20-24s, attendance was higher for women than for men, a difference driven by face-to-face appointments.

Discussion

Summary of results

In England's NHS, CYP outpatient appointments fell sharply in March 2020 and had still not recovered to pre-pandemic levels in March 2021. In total, the 12 months from March 2020 saw 3.8 million appointments fewer (23.5%) than expected from the average for 2017-2019. The fall affected all ages, sexes, specialties (categorised as surgical or non-surgical), and deprivation quintiles. The huge rise in telephone use did not compensate for the bigger fall in face-to-face visits. Around one in five appointments were by phone for this population at the end of the study period.

Attendance rates were high and were higher for phone appointments than for face-to-face ones; the only sex difference was that women aged 20-24 had higher rates than men; for first phone appointments, this sex difference disappeared and was much reduced for follow-ups. In contrast to research showing persistent and systemic child health inequalities in the UK (22), there was little variation in appointment numbers by Index of Multiple Deprivation quintile.

Findings compared with previous studies

Our findings fit with other national research that demonstrated large reductions in secondary care health service use since March 2020 (6,9), including reduced outpatient activity for all age groups (17) Whereas total suspected cancer referrals returned to pre-pandemic levels by Oct 2020, we found that CYP outpatient activity had not returned to pre-pandemic levels at any point by Mar 2021.

Since the Covid-19 pandemic, there has been a significant rise in virtual consultations in CYP outpatient departments globally. A large global survey of healthcare professionals caring for CYP with diabetes found that 50% had switched to virtual consultations (23), 36 of 38 surveyed North American Paediatric surgical units had switched to virtual outpatient appointments (24), a global survey of paediatric neurologists reported a large increase in outpatient virtual consultations (25) and a multicentre longitudinal observational study of Paediatric orthopaedic trauma in London also described greater use of outpatient virtual consultations (26).

Previous studies have also found that in CYP outpatient clinics, telephone appointment attendance is higher than face-to-face appointment attendance (27,28). Studies have shown high levels of patient satisfaction with telephone appointments during the Covid-19 pandemic (29–31) and high levels of satisfaction amongst HCPs providing a remote CYP neurology outpatient service (32). However, concerns have been raised about the quality of

For peer review only - http://bmjopen.bmj.com/site/about/guidelines.xhtml

care provided in remote consultations, especially for first appointments (33,34). Research is emerging on how remote CYP outpatient appointments should best be routinely integrated into post-Covid services (35–37).

The fall in CYP outpatient activity reflects changes in need, a decrease in health seeking behaviour and changes in health system pathways, including a move towards remote consultations and redeployment of Paediatric staff to care for Covid-19 patients. Similar changes in activity during this period were seen across the health system, including in primary care (38) and emergency care (4,39).

The extent to which the fall in CYP outpatient activity can be attributed to a decrease in health seeking behaviour is unknown. A retrospective national cohort study into the decrease of Paediatric secondary care emergency presentations hypothesised that, as there were no increases in clinical severity scores, Paediatric Intensive Care Unit (PICU) admissions or mortality, caregivers were often appropriately able to use a higher threshold for seeking medical attention (6). Another study on this topic also found that 93.5% of CYP presenting to secondary care during a two-week period in April-May 2020 were not felt to have had a delay in their presentation (4). Further research is required to establish the extent to which the fall in CYP outpatient activity since March 2020 is a result of changes in health seeking behaviour, although it is unlikely to have played as large a role as in emergency presentations as we observed a dramatic fall in both first and follow up appointments.

Strengths and limitations

This is the largest study of outpatient appointment patterns in CYP before and after March 2020 in England. It benefits from national data collected in a standardised way. Data quality in such databases for administrative items like dates, age and sex is very high, but we were unable to analyse patterns by diagnosis due to very low levels of recording. Rather than pre and post comparisons, a more sophisticated analysis could be run using interrupted or other time series models, but we inspected pre-pandemic patterns and discerned no clear trends that needed adjustment.

We examined several patient subgroups, but future work could look at patterns by hospital trust and specialty. We saw falls in first and follow-up visits, and one could track whether clinic discharge rates changed during the period. In particular, it will be important to follow patients up to observe their future outcomes, although linkage with primary care records would be needed to capture patients who were not referred to outpatient departments.

Implications for policy and practice

It is reassuring that despite huge changes in clinical practice, face-to-face appointments for babies under 1 year were relatively preserved, compared with the decrease in face-to-face appointments for other age groups, and that activity changes were similar among CYP living in more and less deprived areas. This suggests that the NHS continued to provide a

For peer review only - http://bmjopen.bmj.com/site/about/guidelines.xhtml

responsive, equitable and service for CYP needing specialist care throughout the pandemic, albeit at a reduced level.

While part of the reduction in activity may be accounted for by reduced prevalence of infective conditions and greater parental empowerment and confidence in managing self-limiting illness (4,40), there is likely to be significant ongoing unmet need for specialist paediatric care. Whereas the impact of delay in accessing acute care can be assessed fairly quickly using routinely collected data, the impact of reduced access to outpatient services may be reflected more in worse quality of life for CYP and potentially worse longer term outcomes through lack of early diagnosis and early intervention (41). Identifying and addressing these unmet needs should be a priority for cilnicians, commissioners and policy makers.

These findings may also usefully inform the current debate about integrated care for CYP and the best ways of combining high quality care with convenience and positive experience for patients and families. In particular, greater use of remote care and patient family-initiated follow-up have huge potential benefits as well as potential risks in lower quality care or increasing inequalities. Further research is needed to understand and quantify these risks to guide mitigation strategies.

For peer review only

Conclusions

The impact on patients and the NHS of such a huge fall from pre-pandemic levels in outpatient appointments, including those in surgical specialties, is yet to be felt. The extent to which the fall in total appointments and the rise in the use of telephones matters in terms of patient health outcomes will need further monitoring. The natural experiment of radical changes to paediatric outpatient care during the pandemic period may also inform development of more integrated and responsive specialist services for CYP in the future.

Acknowledgments

Contributorship statement

AB and DH devised the study. AB devised the approach to and conducted the data analysis. FN and AB drafted the manuscript. All authors (AB, FN, KF, RV, SK, PA, SS and DH) contributed to the interpretation of the results and critical revision of the manuscript and approved the final draft.

Competing interests

None declared.

Funding

This study was funded by the National Institute for Health Research grant number NIHR202322 'Understanding the disruption of children and young people's health and healthcare use during and after COVID-19 to inform healthcare and policy responses.' The Dr Foster Unit is an academic unit in the Department of Primary Care and Public Health, within the School of Public Health, Imperial College London. The unit received research funding from Dr Foster Intelligence, an independent health service research organization (a wholly owned subsidiary of Telstra), until September 2021. The Dr Foster Unit at Imperial is affiliated with the National Institute of Health Research (NIHR) Imperial Patient Safety Translational Research Centre. The NIHR Imperial Patient Safety Translational Centre is a partnership between the Imperial College Healthcare NHS Trust and Imperial College London. The Department of Primary Care & Public Health at Imperial College London is grateful for support from the NW London NIHR Applied Research Collaboration and the Imperial NIHR Biomedical Research Centre. The views expressed in this publication are those of the authors and not necessarily those of the NIHR or the Department of Health and Social Care.

Data sharing statement

The pseudonymised patient data that were used for this study can be accessed by contacting NHS Digital (see https://digital.nhs.uk/services/data-access-request-service-dars). Access to these data is subject to a data sharing agreement (DSA) containing detailed terms and conditions of use following protocol approval from NHS Digital.

References

1. Isba R, Edge R, Jenner R, Broughton E, Francis N, Butler J. Where have all the children gone? Decreases in paediatric emergency department attendances at the start of the COVID-19 pandemic of 2020. Arch Dis Child. 2020 Jul 1;105(7):704–704.

2. Scaramuzza A, Tagliaferri F, Bonetti L, Soliani M, Morotti F, Bellone S, et al. Changing admission patterns in paediatric emergency departments during the COVID-19 pandemic. Arch Dis Child. 2020 Jul 1;105(7):704–6.

3. Rose K, Zyl KV, Cotton R, Wallace S, Cleugh F. Paediatric Attendances and Acuity in the Emergency Department during the COVID-19 Pandemic. medRxiv. 2020 Aug 11;2020.08.05.20168666.

4. Roland D, Harwood R, Bishop N, Hargreaves D, Patel S, Sinha I. Children's emergency presentations during the COVID-19 pandemic. Lancet Child Adolesc Health. 2020 Aug 1;4(8):e32–3.

For peer review only

5. McDonnell T, Nicholson E, Conlon C, Barrett M, Cummins F, Hensey C, et al. Assessing the Impact of COVID-19 Public Health Stages on Paediatric Emergency Attendance. Int J Environ Res Public Health [Internet]. 2020 Sep [cited 2021 May 6];17(18). Available from: https://www.ncbi.nlm.nih.gov/pmc/articles/PMC7558983/

6. Williams TC, MacRae C, Swann OV, Haseeb H, Cunningham S, Davies P, et al. Indirect effects of the COVID-19 pandemic on paediatric healthcare use and severe disease: a retrospective national cohort study. Arch Dis Child. 2021 Jan 15;

7. Gill PJ, Mahant S, Hall M, Berry JG. Reasons for Admissions to US Children's Hospitals During the COVID-19 Pandemic. JAMA. 2021 Apr 27;325(16):1676–9.

8. Dann L, Fitzsimons J, Gorman KM, Hourihane J, Okafor I. Disappearing act: COVID-19 and paediatric emergency department attendances. Arch Dis Child. 2020 Aug 1;105(8):810–1.

9. Wyatt S, Mohammed MA, Fisher E, McConkey R, Spilsbury P. Impact of the SARS-CoV-2 pandemic and associated lockdown measures on attendances at emergency departments in English hospitals: A retrospective database study. Lancet Reg Health – Eur [Internet]. 2021 Mar 1 [cited 2021 May 7];2. Available from: https://www.thelancet.com/journals/lanepe/article/PIIS2666-7762(21)00011-9/abstract

10. Creese H, Taylor-Robinson D, Saglani S, Saxena S. Primary care of children and young people with asthma during the COVID-19 era. Br J Gen Pract. 2020 Nov 1;70(700):528–9.

11. Sheath C, Abdelrahman M, MacCormick A, Chan D. Paediatric appendicitis during the COVID-19 pandemic. J Paediatr Child Health. 2021 Feb 1;

12. Gajaweera H, Oladele D, Connett G. Evaluation of the impact of shielding to avoid COVID-19 infection on respiratory symptoms in children with severe asthma. Arch Dis Child. 2021 Apr;106(4):e23.

13. Research Network PST, Arthur F, Harwood R, Allin B, Bethell GS, Boam T, et al. Lockdown during the COVID-19 pandemic: impact on infants with pyloric stenosis. Arch Dis Child. 2020 Dec 1;

14. Diabetes C 19 PU based IG of CC and, Williams G, McLean R, Liu JF, Ritzmann T, Dandapani M, et al. A multi-centre service evaluation of the impact of the COVID-19 pandemic on presentation of newly diagnosed cancers and type 1 diabetes in children in the UK. medRxiv. 2021 Feb 21;2021.02.09.21251149.

15. Langan D, Shelmerdine S, Taylor A, Bryant WA, Booth J, Sebire NJ, et al. Impact of the COVID-19 pandemic on radiology appointments in a tertiary children's hospital: a retrospective study. BMJ Paediatr Open. 2021 Aug 1;5(1):e001210.

16. British Medical Association. The hidden impact of COVID-19 on patient care in the NHS in England [Internet]. 2020 Jul. Available from: https://www.bma.org.uk/media/2841/the-hidden-impact-of-covid_web-pdf.pdf

For peer review only - http://bmjopen.bmj.com/site/about/guidelines.xhtml

17. Thorlby R, Fraser C, Gardner T. Non-COVID-19 NHS care during the pandemic | The Health Foundation [Internet]. [cited 2021 May 7]. Available from: https://www.health.org.uk/news-and-comment/charts-and-infographics/non-covid-19-nhs-care-during-the-pandemic

18. Ruzangi J, Blair M, Cecil E, Greenfield G, Bottle A, Hargreaves DS, et al. Trends in healthcare use in children aged less than 15 years: a population-based cohort study in England from 2007 to 2017. BMJ Open. 2020 May 1;10(5):e033761.

19. Galen LS van, Car J. Telephone consultations. BMJ. 2018 Mar 29;360:k1047.

20. Vaona A, Pappas Y, Grewal RS, Ajaz M, Majeed A, Car J. Training interventions for improving telephone consultation skills in clinicians. Cochrane Database Syst Rev. 2017 Jan 5;1:CD010034.

21. Armstrong S. Universal access to the digital NHS—but only if you have a smartphone. BMJ. 2021 Jul 15;374:n1732.

22. Jessiman PE, Powell K, Williams P, Fairbrother H, Crowder M, Williams JG, et al. A systems map of the determinants of child health inequalities in England at the local level. PloS One. 2021 Feb 12;16(2):e0245577–e0245577.

23. Elbarbary NS, Dos Santos TJ, de Beaufort C, Agwu JC, Calliari LE, Scaramuzza AE. COVID-19 outbreak and pediatric diabetes: Perceptions of health care professionals worldwide. Pediatr Diabetes. 2020 Nov;21(7):1083–92.

24. Ingram MCE, Raval MV, Newton C, Lopez ME, Berman L. Characterization of initial North American pediatric surgical response to the COVID-19 pandemic. J Pediatr Surg. 2020 Aug 1;55(8):1431–5.

25. Wirrell EC, Grinspan ZM, Knupp KG, Jiang Y, Hammeed B, Mytinger JR, et al. Care Delivery for Children With Epilepsy During the COVID-19 Pandemic: An International Survey of Clinicians. J Child Neurol. 2020 Nov;35(13):924–33.

26. Sugand K, Park C, Morgan C, Dyke R, Aframian A, Hulme A, et al. Impact of the COVID-19 pandemic on paediatric orthopaedic trauma workload in central London: a multi-centre longitudinal observational study over the ‘golden weeks’. Acta Orthop. 2020 Dec;91(6):633–8.

27. Duncan H, Russell RK. Role for structured telephone clinics in paediatric gastroenterology: reflections, lessons and patient feedback. BMJ Open Gastroenterol. 2019;6(1):e000245.

28. O’Brien G, Lazebnik R. Telephone call reminders and attendance in an adolescent clinic. Pediatrics. 1998 Jun;101(6):E6.

29. Leibar Tamayo A, Linares Espinós E, Ríos González E, Trelles Guzmán C, Álvarez-Maestro M, de Castro Guerín C, et al. Evaluation of teleconsultation system in the urological patient during the COVID-19 pandemic. Actas Urol Esp. 2020 Nov;44(9):617–22.

For peer review only

30. Darr A, Senior A, Argyriou K, Limbrick J, Nie H, Kantczak A, et al. The impact of the coronavirus (COVID-19) pandemic on elective paediatric otolaryngology outpatient services - An analysis of virtual outpatient clinics in a tertiary referral centre using the modified paediatric otolaryngology telemedicine satisfaction survey (POTSS). Int J Pediatr Otorhinolaryngol. 2020 Nov;138:110383.

31. Wolthers TO, Wolthers OD. Telephone consultation as a substitute for face-to-face consultation during the COVID-19 pandemic. Dan Med J. 2020 Jun 23;67(7).

32. Rametta SC, Fridinger SE, Gonzalez AK, Xian J, Galer PD, Kaufman M, et al. Analyzing 2,589 child neurology telehealth encounters necessitated by the COVID-19 pandemic. Neurology. 2020 Sep 1;95(9):e1257–66.

33. Shaw S, Wherton J, Vijayaraghavan S, Morris J, Bhattacharya S, Hanson P, et al. Advantages and limitations of virtual online consultations in a NHS acute trust: the VOCAL mixed-methods study. Health Serv Deliv Res. 2018 Jun 22;6(21):162.

34. Badawy SM, Radovic A. Digital Approaches to Remote Pediatric Health Care Delivery During the COVID-19 Pandemic: Existing Evidence and a Call for Further Research. JMIR Pediatr Parent. 2020 Jun 25;3(1):e20049–e20049.

35. Charnell AM, Hoen L ’t, Sforza S, Spinoit AF, Radford A. Remote consultations in paediatric urology-Not just for pandemics? J Pediatr Urol. 2021 Apr;17(2):260–2.

36. Cohen E, Cohen MI. COVID-19 will forever change the landscape of telemedicine. Curr Opin Cardiol. 2021 Jan;36(1):110–5.

37. Chen Y, Kathirithamby DR, Li J, Candelario-Velazquez C, Bloomfield A, Ambrose AF. Telemedicine in the Coronavirus Disease 2019 Pandemic: A Pediatric Rehabilitation Perspective. Am J Phys Med Rehabil. 2021 Apr;100(4):321–6.

38. Foley KA, Maile EJ, Bottle A, Neale FK, Viner R, Kenny SE, et al. Impact of covid-19 on primary care contacts with children and young people aged 0-24 years in England; longitudinal trends study 2015-2020. Br J Gen Pract. 2022 Apr 4;BJGP.2021.0643.

39. McConkey R, Hargreaves D, Wyatt S. How has children and young people’s usage of A&E been impacted by lockdown and social distancing? [Internet]. The Health Foundation. 2020. Available from: https://www.health.org.uk/news-and-comment/charts-and-infographics/how-has-children-and-young-peoples-usage-of-AE-been-impacted-by-lockdown-and-social-distancing

40. Chang AY, Cullen MR, Harrington RA, Barry M. The impact of novel coronavirus COVID-19 on noncommunicable disease patients and health systems: a review. J Intern Med. 2020/10/27 ed. 2021 Apr;289(4):450–62.

41. Hargreaves DS, Elliott MN, Viner RM, Richmond TK, Schuster MA. Unmet Health Care Need in US Adolescents and Adult Health Outcomes. Pediatrics. 2015 Sep 1;136(3):513–20.

For peer review only

For peer review only

For peer review only - http://bmjopen.bmj.com/site/about/guidelines.xhtml

Figure Legends

Figure 1. All scheduled appointments since Jan 2018 by age group

Figure 2. Trends in total appointments by IMD fifth for ages 0-1 and 20-24; IMD 1 is least deprived, IMD 5 is most deprived

For peer review only

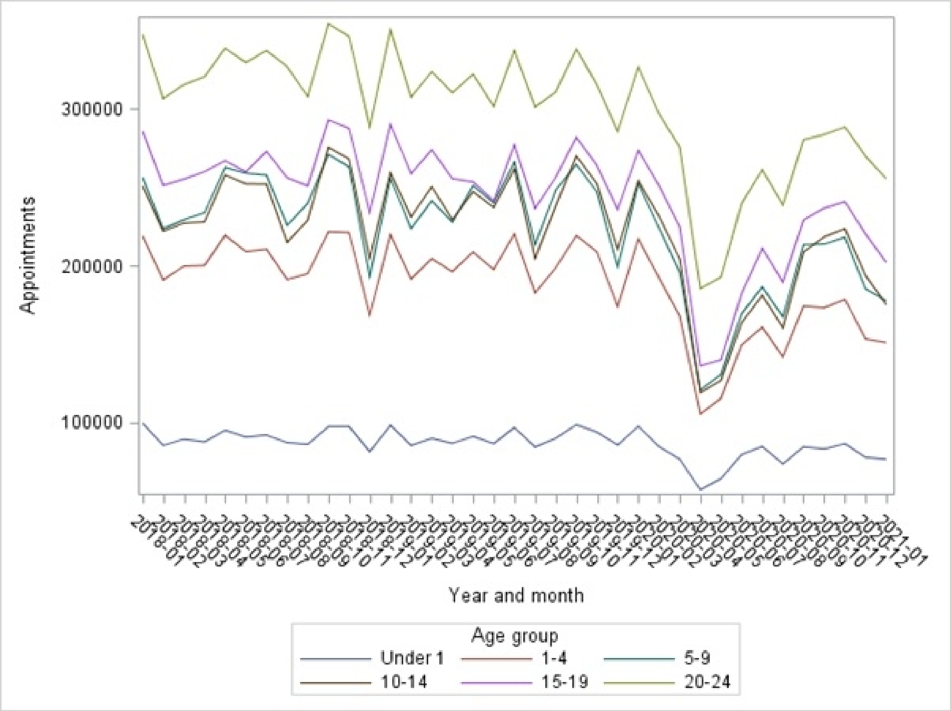

Figure 1. All scheduled appointments since Jan 2018 by age group

53x40mm (300 x 300 DPI)

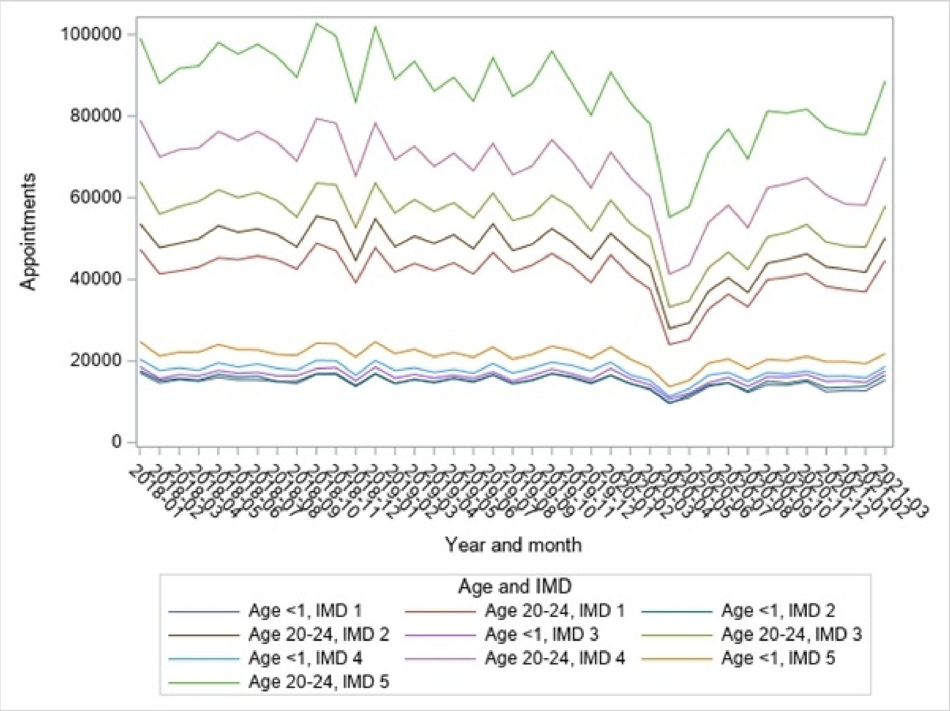

Figure 2. Trends in total appointments by IMD fifth for ages 0-1 and 20-24; IMD 1 is least deprived, IMD 5 is most deprived

53x40mm (300 x 300 DPI)

Supplementary material

Figure S1. All scheduled appointments since Jan 2018 by sex for three age groups. Poisson regression demonstrated a negligible monthly linear trend 2017-2019.

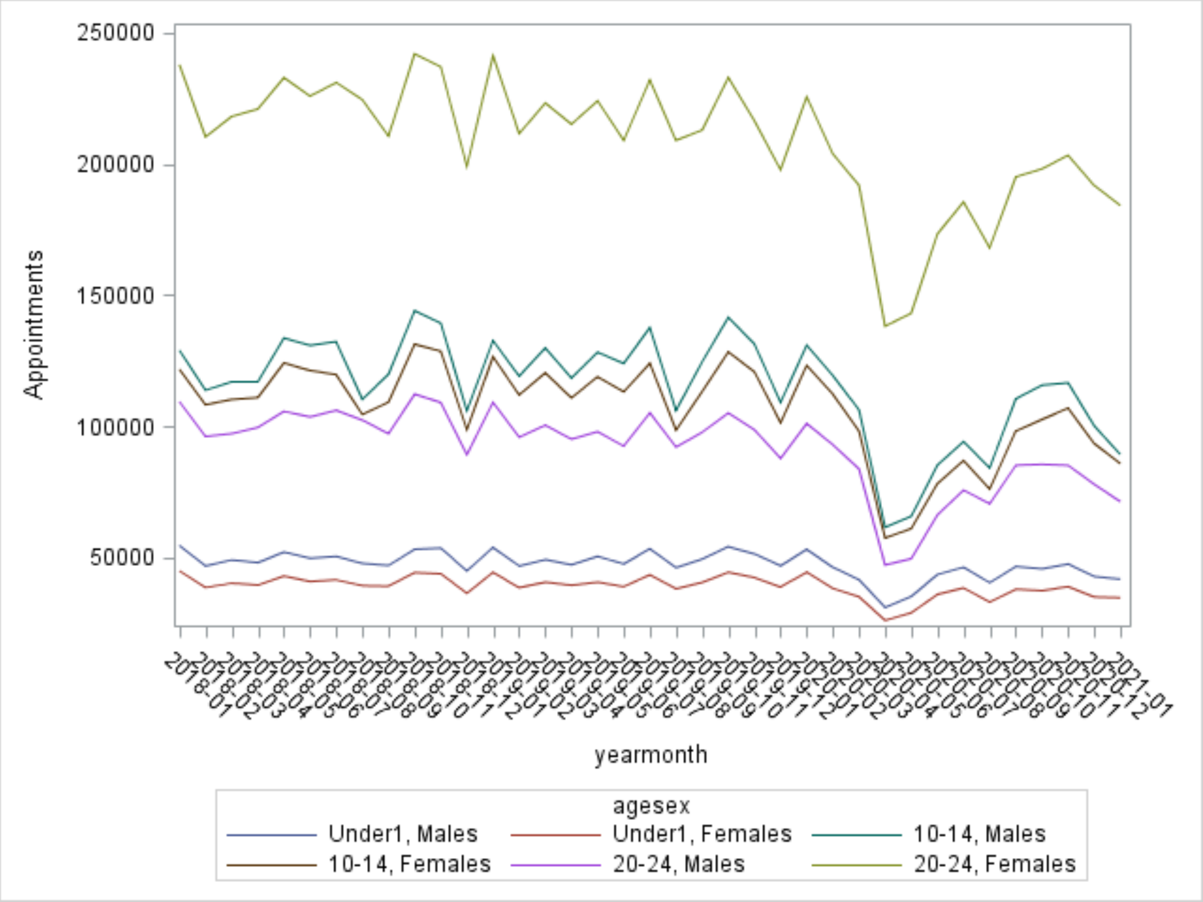

only

Figure S2. All appointments, all ages, split by whether the specialty was surgical

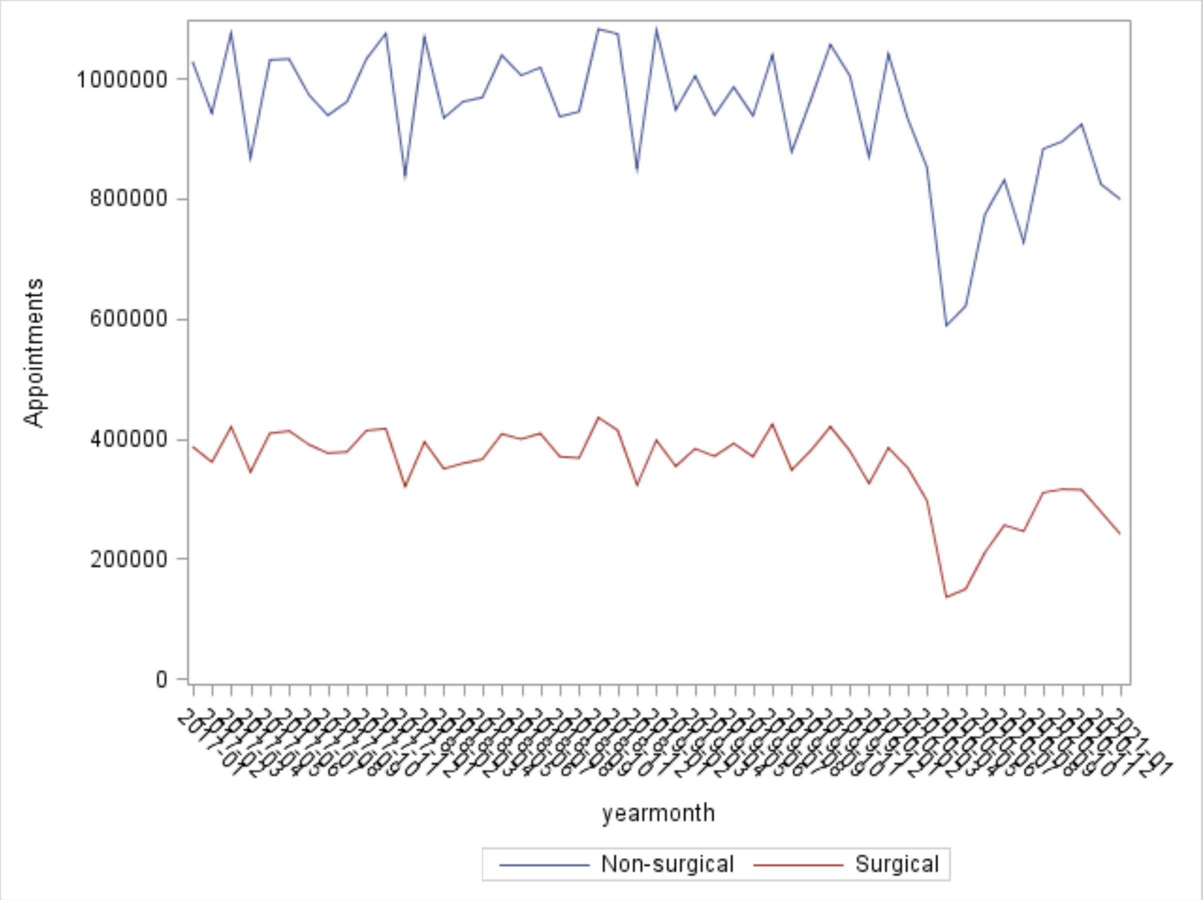

For peer review only

Figure S3. Trends in first appointments (both face-to-face and telephone) by IMD fifth; IMD 1 is least deprived, IMD 5 most deprived

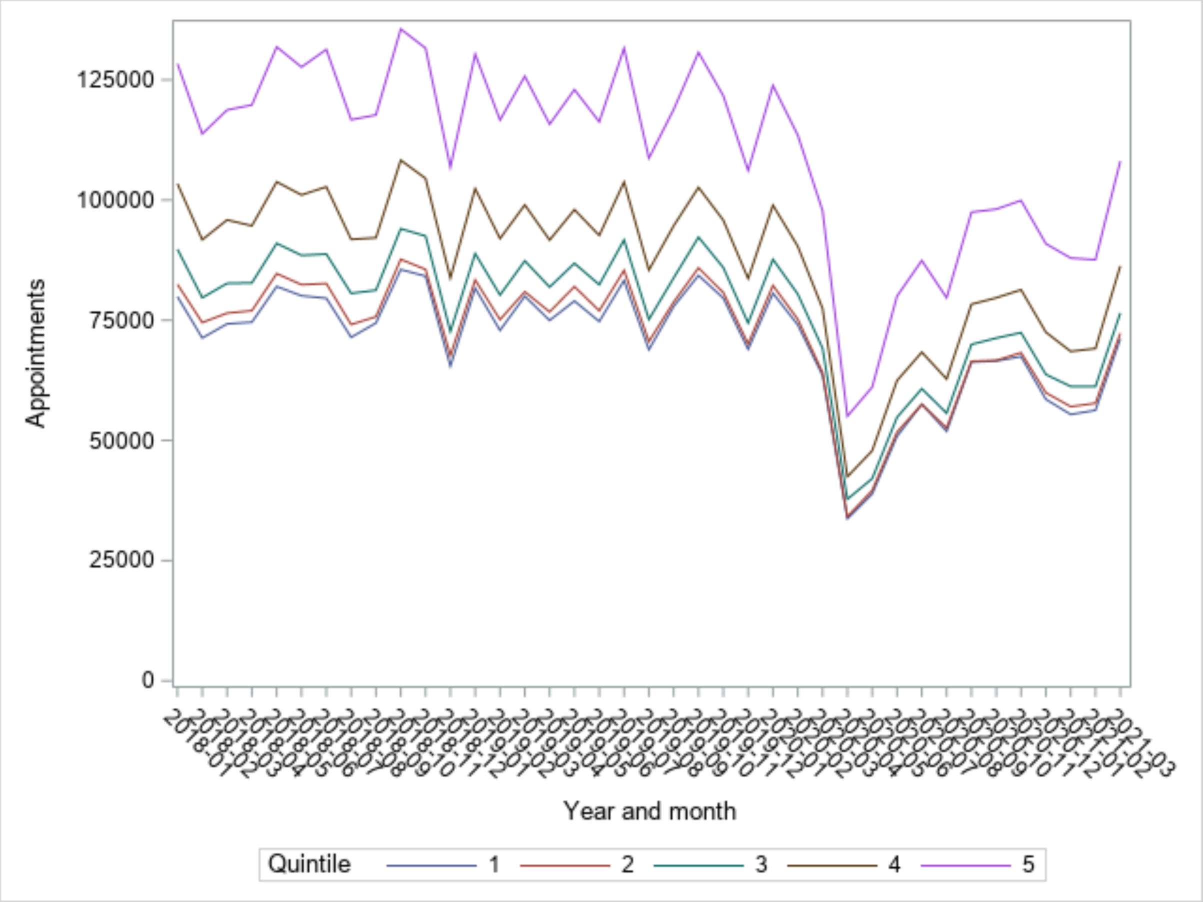

Figure S4. Trends in follow up appointments (both face-to-face and telephone) by IMD fifth; IMD 1 is least deprived, IMD 5 most deprived

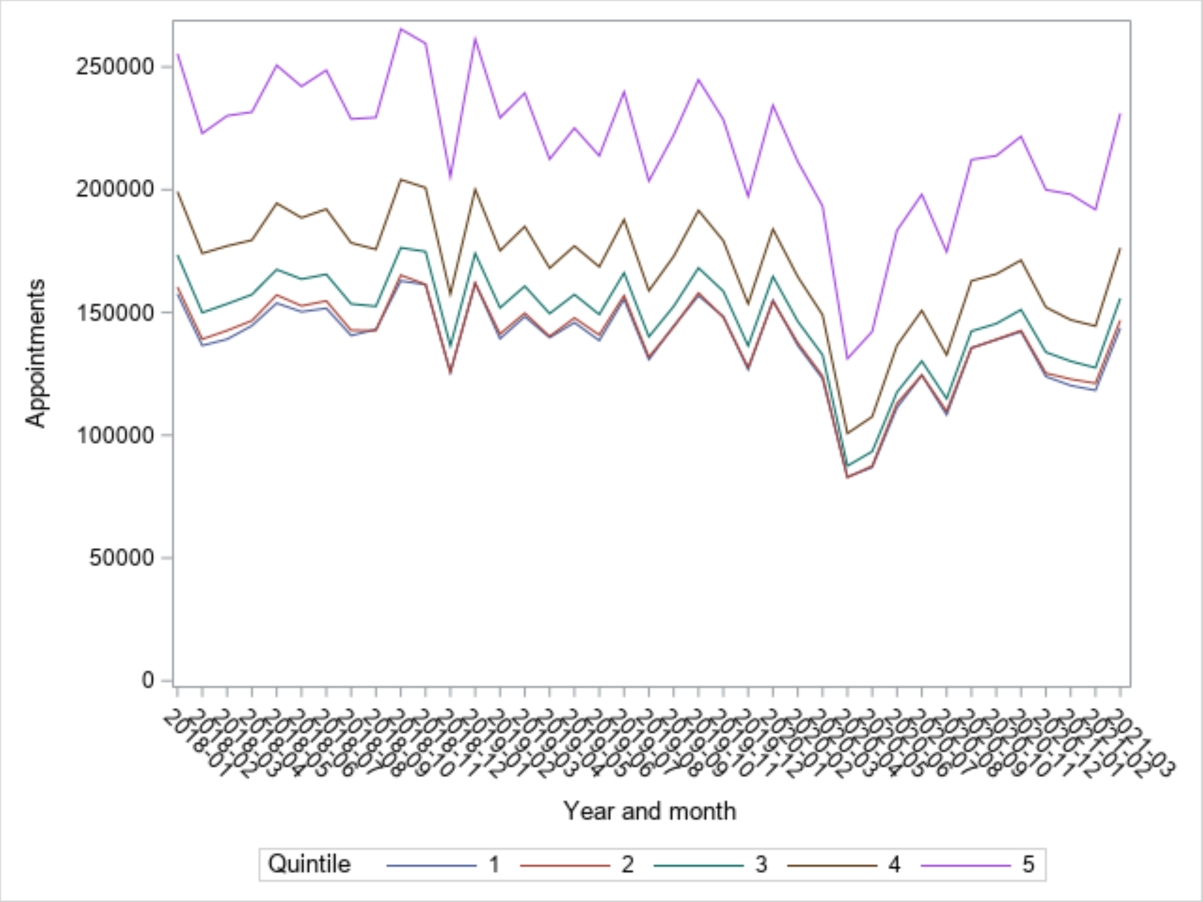

Table S1. Total number of first and follow up appointments face-to-face (F2F) or by telephone, stratified by age and period.

	Jan 2017 to Dec 2019 average				Oct 2020 to Feb 2021 average*			
Age (years)	First appt F2F	First appt by phone	Follow up appt F2F	Follow up appt by phone	First appt F2F	First appt by phone	Follow up appt F2F	Follow up appt by phone
Under-1	595004	9254	459800	22274	427898	59443	335055	91541
1-4	875210	11806	1490240	47186	467160	115895	854808	365760
5-9	893452	20157	1904357	61848	469174	123935	1084465	457184
10-14	862405	30844	1879837	65696	458642	128777	1093129	450506
15-19	916698	28264	2115660	68580	528163	144721	1279882	452514
20-14	1328937	30532	2428495	69945	831865	190481	1548571	447965
Total	5471706	130857	10278389	335529	3182902	763252	6195910	2265470

* Oct 2020 to Feb 2021 is given because the first wave of covid-19 had ended, and the system had stabilised

For peer review only

Figure S5. Increase compared with three-year average for 1st phone appointments

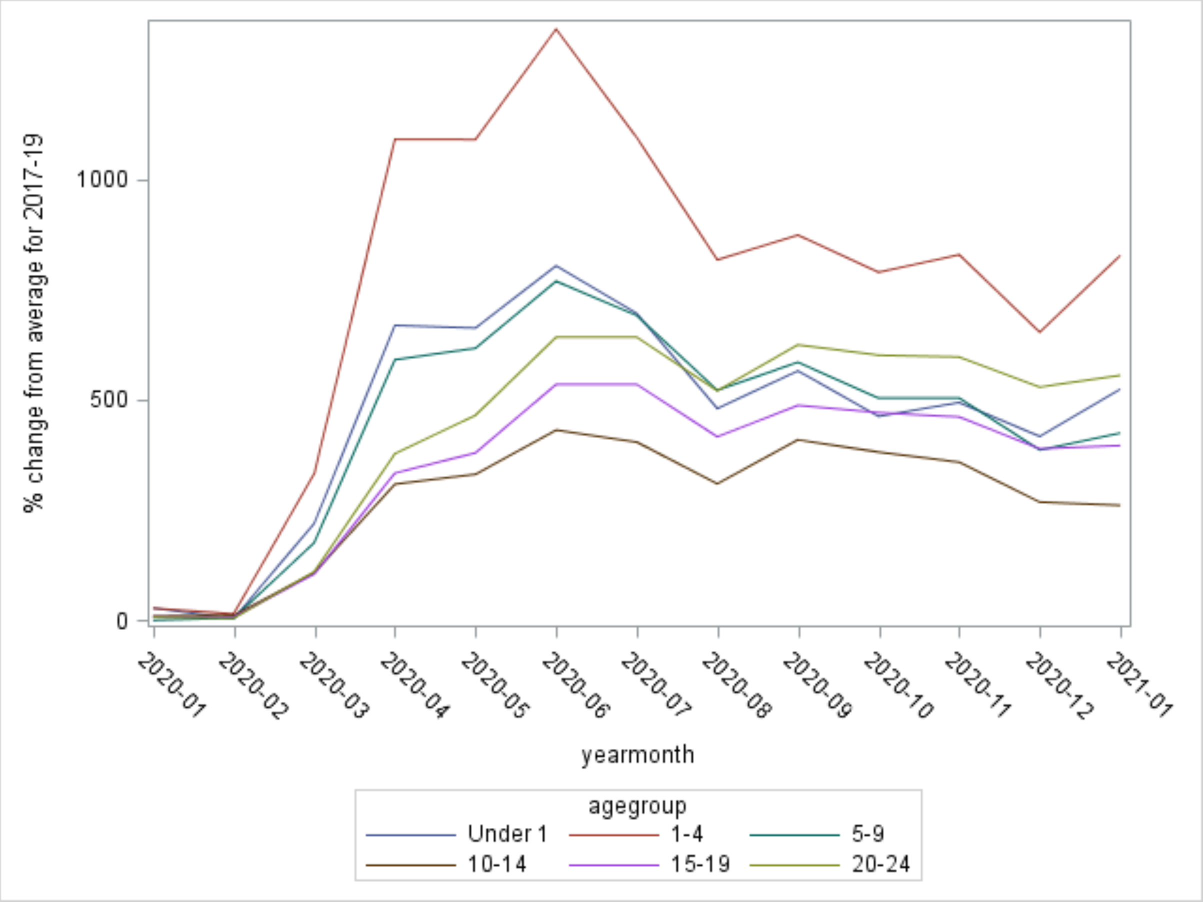

iew only

Figure S6. Attendance rates for the under-1s, comparing F2F with phone appts

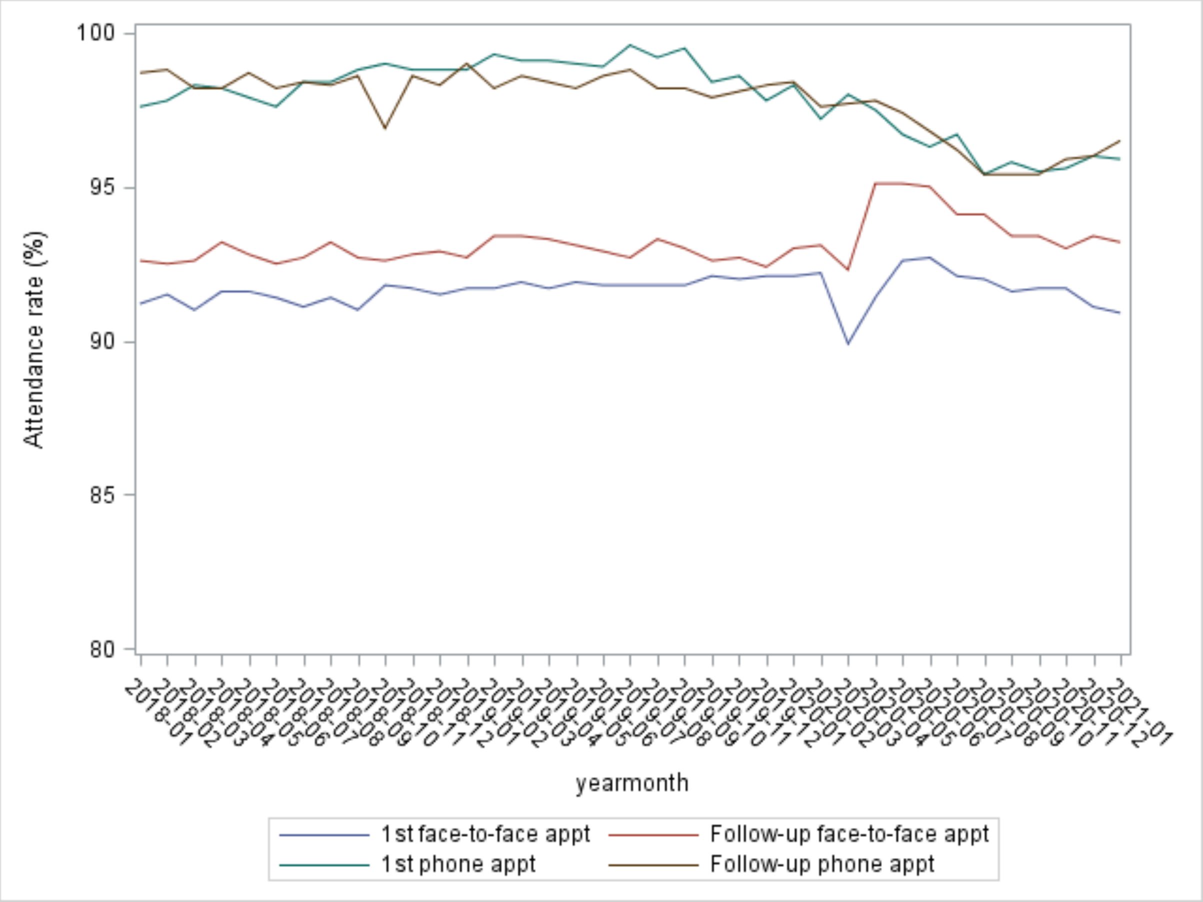

iew only

The pattern is repeated for the other age groups. For example, here are the attendance rates for those aged 20-24:

Figure S7. Attendance rates for the 20-24s, comparing F2F with phone appts

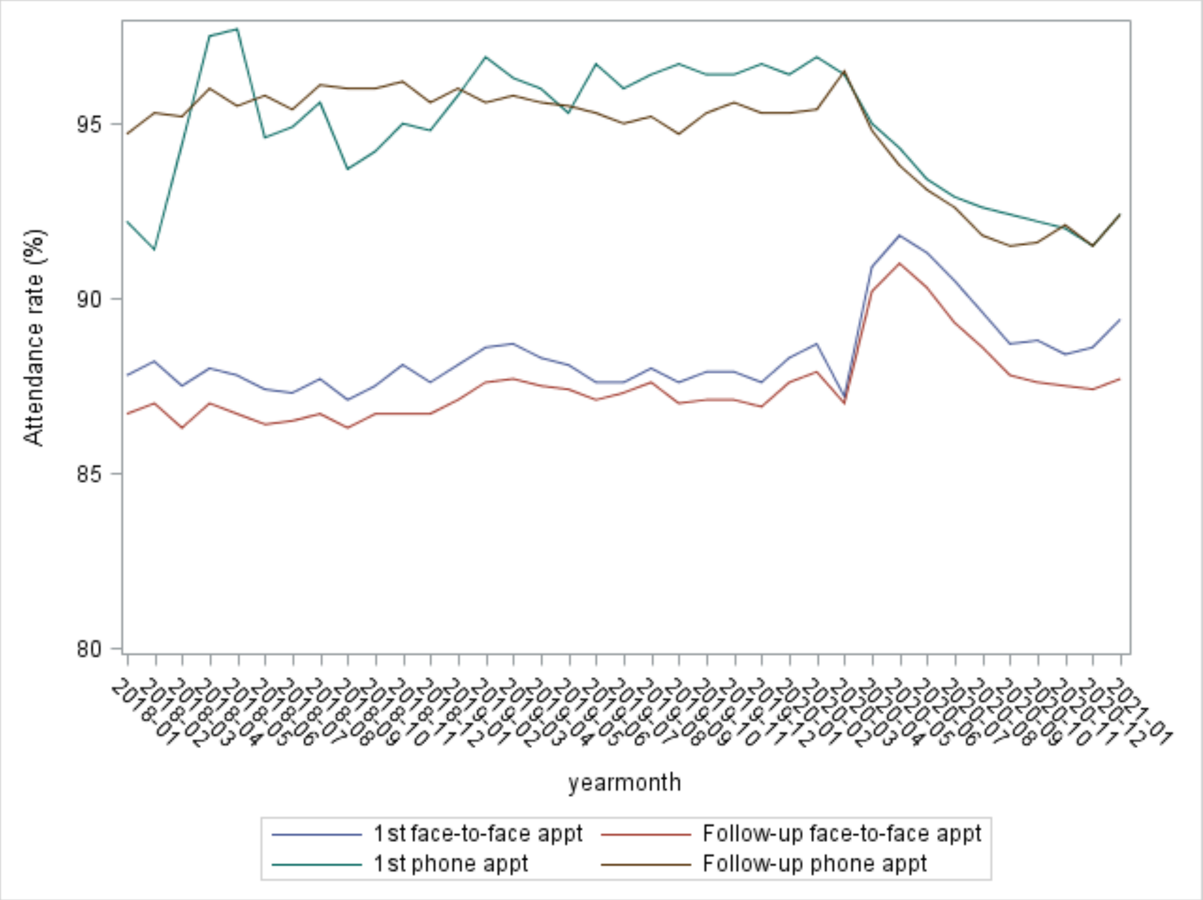

STROBE 2007 (v4) checklist of items to be included in reports of observational studies in epidemiology*
Checklist for cohort, case-control, and cross-sectional studies (combined)

Section/Topic	Item #	Recommendation	Reported on page #
**Title and abstract**	1	(*a*) Indicate the study's design with a commonly used term in the title or the abstract	1
		(*b*) Provide in the abstract an informative and balanced summary of what was done and what was found	1
**Introduction**			
Background/rationale	2	Explain the scientific background and rationale for the investigation being reported	2
Objectives	3	State specific objectives, including any pre-specified hypotheses	2
**Methods**			
Study design	4	Present key elements of study design early in the paper	3
Setting	5	Describe the setting, locations, and relevant dates, including periods of recruitment, exposure, follow-up, and data collection	3
Participants	6	(*a*) *Cohort study*—Give the eligibility criteria, and the sources and methods of selection of participants. Describe methods of follow-up<br>*Case-control study*—Give the eligibility criteria, and the sources and methods of case ascertainment and control selection. Give the rationale for the choice of cases and controls<br>*Cross-sectional study*—Give the eligibility criteria, and the sources and methods of selection of participants	3
		(*b*) *Cohort study*—For matched studies, give matching criteria and number of exposed and unexposed<br>*Case-control study*—For matched studies, give matching criteria and the number of controls per case	
Variables	7	Clearly define all outcomes, exposures, predictors, potential confounders, and effect modifiers. Give diagnostic criteria, if applicable	3
Data sources/ measurement	8*	For each variable of interest, give sources of data and details of methods of assessment (measurement). Describe comparability of assessment methods if there is more than one group	*3*
Bias	9	Describe any efforts to address potential sources of bias	3
Study size	10	Explain how the study size was arrived at	3
Quantitative variables	11	Explain how quantitative variables were handled in the analyses. If applicable, describe which groupings were chosen and why	3
Statistical methods	12	(*a*) Describe all statistical methods, including those used to control for confounding	3
		(*b*) Describe any methods used to examine subgroups and interactions	3
		(*c*) Explain how missing data were addressed	3
		(*d*) *Cohort study*—If applicable, explain how loss to follow-up was addressed<br>*Case-control study*—If applicable, explain how matching of cases and controls was addressed	N/A

For peer review only

		Cross-sectional study—If applicable, describe analytical methods taking account of sampling strategy	
		(*e*) Describe any sensitivity analyses	N/A
**Results**			
Participants	13*	(a) Report numbers of individuals at each stage of study—eg numbers potentially eligible, examined for eligibility, confirmed eligible, included in the study, completing follow-up, and analysed	3
		(b) Give reasons for non-participation at each stage	N/A
		(c) Consider use of a flow diagram	N/A
Descriptive data	14*	(a) Give characteristics of study participants (eg demographic, clinical, social) and information on exposures and potential confounders	4
		(b) Indicate number of participants with missing data for each variable of interest	N/A
		(c) *Cohort study*—Summarise follow-up time (eg, average and total amount)	4
Outcome data	15*	*Cohort study*—Report numbers of outcome events or summary measures over time	
		Case-control study—Report numbers in each exposure category, or summary measures of exposure	
		Cross-sectional study—Report numbers of outcome events or summary measures	4
Main results	16	(*a*) Give unadjusted estimates and, if applicable, confounder-adjusted estimates and their precision (eg, 95% confidence interval). Make clear which confounders were adjusted for and why they were included	N/A
		(*b*) Report category boundaries when continuous variables were categorized	4
		(*c*) If relevant, consider translating estimates of relative risk into absolute risk for a meaningful time period	N/A
Other analyses	17	Report other analyses done—eg analyses of subgroups and interactions, and sensitivity analyses	4
**Discussion**			
Key results	18	Summarise key results with reference to study objectives	4-5
Limitations	19	Discuss limitations of the study, taking into account sources of potential bias or imprecision. Discuss both direction and magnitude of any potential bias	6
Interpretation	20	Give a cautious overall interpretation of results considering objectives, limitations, multiplicity of analyses, results from similar studies, and other relevant evidence	6-7
Generalisability	21	Discuss the generalisability (external validity) of the study results	6-7
**Other information**			
Funding	22	Give the source of funding and the role of the funders for the present study and, if applicable, for the original study on which the present article is based	7

*Give information separately for cases and controls in case-control studies and, if applicable, for exposed and unexposed groups in cohort and cross-sectional studies.

Note: An Explanation and Elaboration article discusses each checklist item and gives methodological background and published examples of transparent reporting. The STROBE checklist is best used in conjunction with this article (freely available on the Web sites of PLoS Medicine at http://www.plosmedicine.org/, Annals of Internal Medicine at http://www.annals.org/, and Epidemiology at http://www.epidem.com/). Information on the STROBE Initiative is available at www.strobe-statement.org.

For peer review only
